# SGLT2 inhibitors and risk of distant metastasis in patients with breast cancer and type 2 diabetes: a target trial emulation with biological plausibility analysis

**DOI:** 10.1016/j.eclinm.2026.104117

**Published:** 2026-07-28

**Authors:** Po-Huang Chen, Wei-Cheng Chang, Hong-Jie Jhou, Hsin-Yu Chen, Mao-Hsuan Huang, Cho-Hao Lee

**Affiliations:** a Division of Haematology and Oncology, Department of Internal Medicine, Tri-Service General Hospital, College of Medicine, National Defense Medical University, Taipei, Taiwan; b Department of Oncology, Tri-Service General Hospital, College of Medicine, National Defense Medical University, Taipei, Taiwan; c Department of Ophthalmology, Universal Eye Center, Taipei, Taiwan; d Neurological Institute, Changhua Christian Hospital, Changhua, Taiwan; e Department of Family Medicine, Chi Mei Medical Center, Tainan, Taiwan; f Department of Psychiatry, Cheng Hsin General Hospital, Taipei, Taiwan

**Keywords:** SGLT2 inhibitors, Breast cancer, Distant metastasis, Target trial emulation, Metabolic reprogramming

## Abstract

**Background:**

Sodium-glucose cotransporter 2 (SGLT2) inhibitors exert pleiotropic effects beyond glucose lowering, including potential anticancer properties. Whether SGLT2 inhibitor use is associated with reduced distant metastasis risk in patients with breast cancer and type 2 diabetes remains unknown.

**Methods:**

We conducted a target trial emulation study using the TriNetX Global Collaborative Network (194 million patients; data covering Jan 1, 2017 to Dec 31, 2025). Adult women with breast cancer, mastectomy, and type 2 diabetes who initiated SGLT2 inhibitors (exposure) or other antidiabetic medications (comparator) post-mastectomy were identified using a new-user design. After 1:1 propensity score matching (3569 per group), we assessed composite distant metastasis (ICD-10: C78 + C79) and organ-specific metastasis using 90-day landmark analysis over five-year follow-up. An exploratory transcriptomic analysis using paired primary–metastasis RNA-sequencing data (GSE110590) assessed biological plausibility.

**Findings:**

SGLT2 inhibitor use was associated with a reduction in composite distant metastasis (HR 0·676; 95% CI 0·532–0·859; p = 0·001). Organ-specific analyses showed the strongest protection for liver metastasis (HR 0·461; 95% CI 0·278–0·765; p < 0·001), followed by lung (HR 0·550; 95% CI 0·351–0·863; p < 0·001) and bone metastasis (HR 0·576; 95% CI 0·394–0·843; p < 0·001), while brain metastasis showed no significant association (HR 0·837; 95% CI 0·460–1·523; p = 0·481). Results remained significant in per-protocol and US-only sensitivity analyses. Negative control outcomes confirmed absence of systematic bias. Liver metastases exhibited the broadest metabolic hyperactivity (eight of 15 pathways at FDR<0·05), directionally consistent with organ-specific clinical protection.

**Interpretation:**

SGLT2 inhibitor use was associated with significantly reduced distant metastasis risk, with an organ-specific gradient that was directionally consistent with exploratory transcriptomic findings. These hypothesis-generating observations should be interpreted with caution. Prospective randomised controlled trials with pre-specified organ-specific distant metastasis endpoints and a dedicated post-mastectomy SGLT2 inhibitor initiation protocol are needed to establish whether the observed associations reflect a causal protective effect before any change in clinical practice.

**Funding:**

This study was supported by the Tri-Service General Hospital and by a collaborative research grant between Cheng Hsin General Hospital and National Defense Medical University. The funders had no role in study design, data collection, data analyses, data interpretation, or the writing of the report.

**Research in context**

**Evidence before this study**

We searched PubMed and Embase from Jan 1, 2010, to March 31, 2025, using the terms “SGLT2 inhibitor” AND (“breast cancer” OR “metastasis” OR “cancer outcomes”), with no language restrictions. Preclinical studies demonstrated that SGLT2 inhibitors activate AMPK and inhibit tumour cell proliferation. One observational study reported delayed hormone therapy failure in prostate cancer with SGLT2 inhibitor use. A melanoma mouse model showed reduced liver metastasis with SGLT2 inhibition via disruption of insulin-dependent PI3K/AKT signalling. However, no study had examined the association between SGLT2 inhibitor use and distant metastasis risk in breast cancer, and no investigation had evaluated organ-specific metastasis patterns in this context.

**Added value of this study**

Using a target trial emulation framework with 7138 propensity score-matched patients with breast cancer and type 2 diabetes, we found that SGLT2 inhibitor use was associated with a 32·4% reduction in distant metastasis risk over five years. Protection was organ-specific: strongest for liver and lung metastasis, significant for bone, and non-significant for brain metastasis. An independent exploratory transcriptomic analysis revealed that clinical protection corresponded to the degree of metabolic hyperactivity at each metastatic site, a pattern that is directionally consistent with a plausible biological basis, pending experimental validation.

**Implications of all the available evidence**

These findings suggest that SGLT2 inhibitors, prescribed primarily for diabetes management, might offer secondary anticancer benefits in patients with breast cancer and type 2 diabetes. The organ-specific protection pattern provides a hypothesis-generating framework linking metabolic vulnerability of metastatic niches to drug efficacy. Prospective randomised controlled trials that pre-specify organ-specific distant metastasis endpoints, include both diabetic and non-diabetic breast cancer populations, and integrate paired primary–metastasis molecular profiling are needed to establish whether the observed associations reflect a causal protective effect before any change in clinical practice.

## Introduction

Breast cancer remains the most commonly diagnosed malignancy among women worldwide, with approximately 2·3 million new cases annually.^1^ Despite advances in early detection and multimodal treatment, distant metastasis continues to be the principal cause of breast cancer mortality, with a five-year survival rate of approximately 25% for metastatic disease.^2^ Type 2 diabetes mellitus (T2DM), which affects an estimated 15–20% of patients with breast cancer, has been consistently associated with increased risk of distant recurrence and reduced survival.^3^

Sodium-glucose cotransporter 2 (SGLT2) inhibitors are a class of antidiabetic agents that lower blood glucose by promoting renal glucose excretion.^4^ Beyond glycaemic control, these agents have demonstrated significant cardiovascular and renal protective effects, leading to expanded indications for heart failure and chronic kidney disease regardless of diabetes status.^5^^,^^6^ Emerging evidence suggests that SGLT2 inhibitors might also exert pleiotropic anticancer effects through multiple mechanisms. Canagliflozin activates AMP-activated protein kinase (AMPK) by inhibiting mitochondrial complex I,^7^ while SGLT2 inhibitors more broadly modulate cellular energy metabolism, reduce insulin and insulin-like growth factor 1 (IGF-1) signalling, and attenuate inflammatory pathways.^8^^,^^9^ In preclinical models, canagliflozin inhibited proliferation of hepatocellular carcinoma cells through glucose uptake inhibition,^10^ and SGLT2 inhibition combined with PI3K inhibition reduced liver metastasis in a melanoma mouse model by disrupting the insulin-dependent PI3K/AKT pathway that drives liver-metastatic organotropism.^11^

Recent clinical evidence has begun to translate these preclinical findings. Shi and colleagues reported that SGLT2 inhibitor use was associated with delayed hormone therapy failure in prostate cancer (HR 0·63; 95% CI 0·41–0·95).^12^ However, no study has examined whether SGLT2 inhibitors affect distant metastasis risk in breast cancer, and no prior investigation has evaluated organ-specific metastasis patterns in this context.

The biological plausibility for organ-specific protection is particularly compelling. Breast cancer metastases exhibit distinct metabolic phenotypes depending on the target organ,^13^ and SGLT2 inhibitors, through their effects on glucose availability and energy metabolism, might differentially affect metastatic niches with varying metabolic demands. This organ-specific metabolic hypothesis has not been previously tested.

We therefore conducted this study with two objectives: first, to investigate whether SGLT2 inhibitor use is associated with reduced risk of distant metastasis in patients with breast cancer and T2DM using a target trial emulation framework; and second, to explore the biological plausibility of observed organ-specific patterns through an independent transcriptomic analysis of paired primary tumour and distant metastasis specimens.

## Methods

### Study design and data source

We conducted a retrospective cohort study using a target trial emulation (TTE) framework with propensity score matching. This design emulates a hypothetical randomised controlled trial comparing SGLT2 inhibitor initiation versus other antidiabetic medication initiation among diabetic women following mastectomy for breast cancer. The TTE approach addresses common biases in observational pharmacoepidemiology by explicitly specifying the target trial protocol and aligning eligibility, treatment assignment, and follow-up initiation at a common time zero.^14^^,^^15^

The study utilised electronic health record data from the TriNetX Global Collaborative Network, a federated health research platform encompassing over 194 million patients across 171 healthcare organisations worldwide.^16^ The study period spanned 1 January 2017 to 31 December 2025. This study followed the STROBE guidelines^17^ and the TARGET statement.^18^ The TriNetX platform data is de-identified using the Expert Determination method under HIPAA regulations.

### Ethics

This study was conducted in accordance with the principles of the Declaration of Helsinki and was approved by the Institutional Review Board of Tri-Service General Hospital, National Defense Medical University (TSGHIRB No.: E202516013; approved March 10, 2025) with a Certificate of Review Exemption. The requirement for written informed consent was waived by the Institutional Review Board owing to the retrospective design and the use of de-identified electronic health record data, in accordance with the TriNetX platform's compliance with the HIPAA Expert Determination method.

### Target trial specification

The target trial protocol is detailed in eMethods S1 (Appendix). Briefly, we emulated a trial that would randomise diabetic women with breast cancer, at the time of mastectomy, to initiate either SGLT2 inhibitors or other antidiabetic medications post-operatively. The index date (time zero) was defined as the mastectomy procedure date for all patients. This alignment of eligibility, treatment assignment, and follow-up initiation at a common time zero is essential for valid causal inference and prevents immortal time bias.^19^ Mastectomy was selected as the time-zero anchor because the surgical date is reliably captured in structured records, provides a uniform post-operative reference state (absence of macroscopic primary disease) for counting incident metastasis events, and falls within a well-defined 90-day adjuvant-decision window aligned with the new-user grace period. Restricting to mastectomy also minimises heterogeneity in adjuvant pathways and surveillance intensity that would arise from pooling with breast-conserving-surgery patients.

### Study population and eligibility criteria

Eligibility was assessed at mastectomy (time zero). The study population included adult women (≥18 years) with: (1) a diagnosis of malignant neoplasm of breast (ICD-10-CM: C50), (2) mastectomy procedure (CPT: 19,301–19,307 or equivalent SNOMED codes), (3) type 2 diabetes mellitus (ICD-10-CM: E11) diagnosed before mastectomy, and (4) use of blood glucose-lowering drugs (ATC: A10B) after diabetes diagnosis. Sex was ascertained from structured TriNetX fields as a binary biological variable; gender identity is not captured in the platform, so “sex” here refers to the biological variable. The cohort was sex-restricted to women by the breast cancer (C50) and mastectomy criteria; male breast cancer, accounting for approximately 1% of diagnoses, was not represented.

Patients were excluded if they had: (1) any SGLT2 inhibitor prescription before mastectomy (to ensure new-user status), (2) metastatic disease (ICD-10-CM: C77–C79) within three months before or after mastectomy, (3) outcome events occurring before the index date, or (4) index event occurring more than 20 years before analysis date. The new-user design was employed to minimise prevalent user bias.^20^

### Treatment strategies and exposure definition

The exposure cohort (SGLT2 inhibitor users) included patients whose first antidiabetic prescription after mastectomy was an SGLT2 inhibitor (dapagliflozin, empagliflozin, canagliflozin, or ertugliflozin; ATC: A10BK). The comparator cohort included patients whose first antidiabetic prescription after mastectomy was a non-SGLT2i medication, with no subsequent SGLT2 inhibitor use during follow-up. Drug codes are provided in eTable S9 (Appendix).

### Follow-up and outcome assessment

To avoid immortal time bias, we implemented a 90-day landmark analysis. Follow-up began at Day 90 post-mastectomy, providing a window for treatment initiation while ensuring patients in both groups had equal opportunity to initiate their assigned treatment. Patients were followed from Day 90 until outcome occurrence, death, loss to follow-up, or five years (1825 days post-mastectomy), whichever occurred first.

The primary outcome was a composite distant metastasis endpoint, defined as secondary malignant neoplasm of respiratory and digestive organs (ICD-10-CM: C78) or secondary malignant neoplasm of other and unspecified sites (C79) occurring ≥90 days post-mastectomy. Secondary outcomes included organ-specific distant metastasis: lung (C78·0–C78·3), liver (C78·7), brain (C79·3), and bone (C79·5) metastasis, and bone metastasis with skeletal-related events. Outcome definitions are detailed in eTable S10 (Appendix). Negative control outcomes included traumatic fractures and kidney stones (N20–N23).^21^ To interrogate detection bias from differential abdominal or thoracic imaging, three post-hoc imaging-detected negative control outcomes were also examined: thyroid nodule (E04·1), hepatic cyst (K76·89), and renal cyst (N28·1), incidentally detected through the same imaging modalities used for metastasis staging but with no pharmacological link to SGLT2 inhibitors. Two post-hoc negative control exposure (NCE) analyses tested drug-class specificity: NCE-1 substituted dipeptidyl peptidase-4 (DPP-4) inhibitor as the index exposure to test within-antidiabetic-class confounding, and NCE-2 substituted statin to test general chronic-medication healthy-adherer effects (both NCE comparisons excluded any SGLT2 inhibitor use in either arm). Full negative control design and rationale are detailed in eMethods S4 (Appendix).

### Propensity score matching

Propensity scores were estimated using logistic regression with baseline covariates measured before mastectomy (time zero). Covariates included demographics (age, sex, race, ethnicity), comorbidities (essential hypertension, heart failure, ischaemic heart disease, chronic kidney disease, cerebrovascular diseases, overweight/obesity, dyslipidaemia), cancer treatment history (antineoplastic chemotherapy, antineoplastic hormones, trastuzumab), diabetes medications (metformin, sulfonylureas, alpha-glucosidase inhibitors, thiazolidinediones, DPP-4 inhibitors, GLP-1 receptor agonists, insulin), cardiovascular medications (beta-blockers, ACE inhibitors, ARBs), and laboratory values (HbA1c). Complete covariate definitions are provided in eTable S11 (Appendix). Race and ethnicity were operationalised as US Census Bureau-aligned categories (white, black or african american, asian, and Unknown) self-reported in the contributing electronic health records and aggregated by TriNetX; these categories represent sociocultural constructs rather than biological traits and were used to enable demographic-stratified analyses and to characterise the representativeness of the study population. A 1:1 nearest-neighbour matching was performed using a greedy algorithm with a caliper width of 0·1 standard deviations of the logit propensity score. Covariate balance was assessed using standardised mean differences (SMD), with SMD <0·1 considered adequate balance.^22^^,^^23^

### Statistical analysis

Time-to-event outcomes were analysed using Kaplan–Meier survival analysis with log-rank tests, and hazard ratios (HR) with 95% confidence intervals (CI) were estimated using Cox proportional hazards regression. The primary analysis followed a modified intention-to-treat (mITT) framework in which patients were analysed according to their initial treatment assignment. In the comparator cohort, patients who subsequently initiated an SGLT2 inhibitor during follow-up were excluded at baseline through the new-user design; this aligns the comparator with a per-protocol approach on the comparator side and prevents contamination of the non-SGLT2i cohort. In the SGLT2 inhibitor cohort, patients who later discontinued or switched were retained per intention-to-treat. A post-hoc pure intention-to-treat sensitivity analysis retained comparator-cohort crossovers (eTable S2b, Appendix), and pre-specified per-protocol and US-only sensitivity analyses were also conducted. Given the time-varying pattern observed in the time-dependent analysis, the 5-year Cox hazard ratio is interpreted as a time-averaged summary rather than a constant-over-time effect; formal Schoenfeld diagnostics could not be obtained within the federated platform, so the time-stratified hazard ratios constitute the principal characterisation of effect proportionality. Across the organ-specific, time-stratified, sensitivity, subgroup, and negative-control analyses, no formal family-wise or false-discovery-rate adjustment was applied; these are reported descriptively with 95% confidence intervals and interpreted as supportive of, rather than confirmatory for, the primary composite endpoint. This study was not pre-registered; the analytical protocol is documented in the Appendix (eMethods S1). In accordance with the SAGER reporting guidelines, eligibility was restricted to biologically female adults, consistent with the predominantly female epidemiology of breast cancer and the mastectomy eligibility requirement.

Pre-specified subgroup analyses were performed by age categories (18–49, 50–64, 65–74, ≥75 years), race/ethnicity (white, black or african american, asian), breast cancer molecular subtype (HR+/HER2−, HER2+, triple-negative), and specific SGLT2 inhibitor agent (empagliflozin, dapagliflozin, canagliflozin). P values for interaction were calculated to assess effect modification.

E-values were calculated to assess robustness to unmeasured confounding using the method of VanderWeele and Ding.^24^ Values ≥ 2·0 were interpreted as moderate and ≥3·0 as strong robustness. All cohort-level analyses (Kaplan–Meier survival analysis, log-rank tests, Cox proportional hazards regression, and propensity score matching) were performed on the TriNetX Analytics platform (https://trinetx.com/), which implements survival analyses using R-based libraries (survival package version 3·5–7 current at execution; data extraction for the analyses reported here was performed on 23 May 2026 from the continuously updated live analytics build). The independent exploratory transcriptomic analysis used R version 4·3·1 with the GSVA package (version 1·46·0) for ssGSEA, the stats package for Wilcoxon signed-rank tests and Benjamini-Hochberg correction, and the effsize package for Cohen's d. Sample size was not pre-calculated; the analytic cohort comprised the maximum number of eligible patients within the network at query, as is standard for pharmacoepidemiologic analyses of a fixed population. A two-sided p < 0·05 was considered significant throughout.

As a post-hoc sensitivity analysis to further assess the robustness of the time-dependent pattern observed in the primary 90-day landmark analysis and to more conservatively exclude prevalent undiagnosed metastases at baseline, we repeated the primary analysis using a 180-day landmark. Patients with composite distant metastasis events occurring before Day 180 post-mastectomy were excluded; follow-up began at Day 180. Hazard ratios with 95% confidence intervals were estimated at three pre-specified cumulative time windows: 1-year (D180–D365), 3-year (D180–D1,095), and 5-year (D180–D1,825).

To address potential competing risk from non-cancer mortality, we implemented a post-hoc four-layer strategy. First, all-cause mortality was modelled as a separate endpoint at 1-, 3-, and 5-year windows within the same 1:1 propensity-score-matched cohort. Second, Aalen-Johansen non-parametric cumulative incidence functions for distant metastasis and all-cause mortality, each treated as the competing event for the other, were computed on the unmatched eligible cohorts (the federated platform does not support post-match estimation) and presented as descriptive cross-validation. Third, cause-specific hazard ratios were retained as the primary estimand because the target trial emulation addresses an aetiological question—whether SGLT2 inhibitor use alters the biological rate of distant metastasis. Fourth, formal Fine–Gray subdistribution-hazards regression was not feasible because the federated platform does not support extraction of the individual-level time-to-event data required for covariate-adjusted subdistribution hazard estimation.

### Molecular subtype classification

Molecular subtype was inferred from structured electronic health record data rather than a commercial multi-gene assay or central pathology review. The TriNetX network does not expose underlying assay reports (immunohistochemistry, fluorescence in situ hybridisation, or multi-gene assay results); contributed receptor-status codes are presumed to derive from routine clinical assessment, although assay method and laboratory quality control vary across organisations. We therefore used a pre-specified hierarchical algorithm combining ICD-10-CM receptor-status codes (Z17·0–Z17·421) with post-mastectomy subtype-defining medication exposure (anti-HER2 therapy, endocrine therapy, CDK4/6 inhibitors), assigning patients to HER2-positive, HR-positive/HER2-negative, or triple-negative categories in that order; the full algorithm, code lists, and four acknowledged limitations are detailed in eMethods S3 (Appendix).

### Exploratory transcriptomic analysis for biological plausibility

To explore potential biological mechanisms underlying organ-specific patterns, we performed an independent transcriptomic analysis using publicly available paired primary–metastasis RNA-sequencing data from the GSE110590 dataset.^13^ This dataset contains paired primary tumour and distant metastasis samples from 16 patients with breast cancer across four metastatic sites: liver (n = 10 pairs), lung (n = 10 pairs), brain (n = 6 pairs), and bone (n = 4 pairs).

Pathway activity scores were computed using single-sample gene set enrichment analysis (ssGSEA) as implemented in the GSVA R package.^25^ We assessed 14 biologically relevant gene sets selected *a priori* based on relevance to glucose/energy metabolism and metastatic microenvironment biology (eTable S12, Appendix). Within-patient changes in pathway activity between primary tumour and paired metastasis were assessed using Wilcoxon signed-rank tests, with Benjamini-Hochberg correction for multiple testing. Effect sizes were quantified using paired Cohen's d with 95% confidence intervals computed by normal approximation (d ± 1·96/√n). A composite metabolic demand score was calculated as the mean Cohen's d across five core metabolic pathways (glycolysis, oxidative phosphorylation, fatty acid metabolism, hypoxia, mTORC1 signalling). In brief, single-sample gene set enrichment analysis (ssGSEA) ranks all genes within each tumour sample by their expression level and computes a continuous score quantifying how strongly the genes belonging to a predefined biological pathway are concentrated near the top of that ranking; the paired primary-metastasis design then tests whether metabolic pathway activity in metastatic tissue at each organ site differs systematically from the same patient's primary tumour, thereby controlling for inter-patient heterogeneity. Complete methodological details are provided in eMethods S2 (Appendix).

### Role of the funding source

This study was supported by the Tri-Service General Hospital and by a collaborative research grant between Cheng Hsin General Hospital and National Defense Medical University. The funders had no role in study design, data collection, data analysis, data interpretation, or writing of the report.

## Results

**Fig. 1:**
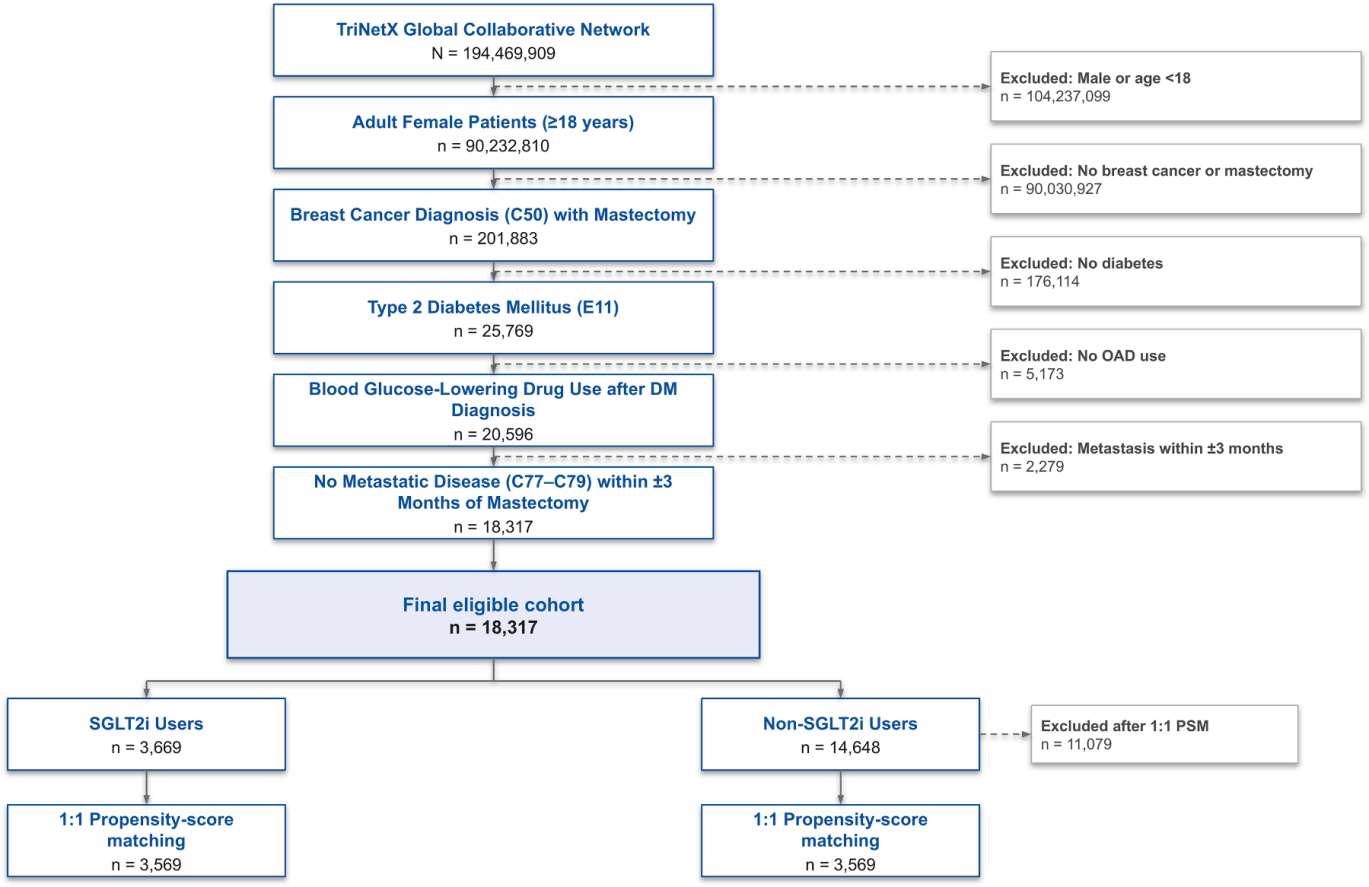
**Study profile.** Sequential application of inclusion and exclusion criteria to derive the final propensity score-matched cohort from the TriNetX Global Collaborative Network using a target trial emulation framework with new-user design. Numbers in parentheses indicate the patient count at each step. After matching, 3569 patients remained in each group. CPT, Current Procedural Terminology code; DM, diabetes mellitus; ICD-10-CM, International Classification of Diseases, 10th Revision, Clinical Modification; OAD, oral antidiabetic drug; PSM, propensity score matching; SGLT2i, sodium-glucose cotransporter 2 inhibitor.

From the TriNetX Global Collaborative Network comprising 194,469,909 patients, sequential application of eligibility criteria identified 18,317 patients meeting all inclusion criteria (Fig. 1). Among these, 3669 were SGLT2 inhibitor users and 14,648 were non-SGLT2 inhibitor users. After 1:1 propensity score matching, 3569 patients remained in each group. Geographically, the eligible patients were drawn from 95 contributing healthcare organisations and were predominantly US-based (13,454 patients; 73·4%), with the remaining 4863 patients (26·6%) contributed by international sites across Asia–Pacific, Europe/Middle East/Africa, Latin America, and other international regions; the complete distribution by US Census Division and international TriNetX region is provided in eTable S15.

Baseline characteristics before and after matching are presented in Table 1. After matching, all covariates achieved adequate balance with SMD <0·1. In the matched cohorts, the mean age was 63·2 ± 10·3 years in the SGLT2 inhibitor group and 63·1 ± 10·8 years in the comparator group (SMD 0·009). The majority of patients were white (64·0% vs 66·1%), followed by black or african american (20·4% vs 18·1%) and asian (6·7% vs 7·2%). Mean HbA1c was 7·2 ± 1·6% versus 7·1 ± 1·6% (SMD 0·023). Comorbidity profiles, cancer treatment history, and medication use were well balanced across groups.

**Table 1:** Baseline characteristics of patients before and after propensity score matching.

Characteristics	Before PSM				After PSM			
	SGLT2i (n = 3669)	Non-SGLT2i (n = 14,648)	p-value	SMD	SGLT2i (n = 3569)	Non-SGLT2i (n = 3569)	p-value	SMD
Demographics								
Age at index, years, mean ± SD	63·2 ± 10·3	63·9 ± 10·8	<0·001	0·067	63·2 ± 10·3	63·1 ± 10·8	0·711	0·009
Race, n (%)								
White	2284 (64·0%)	9498 (66·8%)	0·002	0·059	2283 (64·0%)	2358 (66·1%)	0·063	0·044
Black or african american	727 (20·4%)	2720 (19·1%)	0·093	0·031	727 (20·4%)	646 (18·1%)	0·015	0·058
Asian	238 (6·7%)	831 (5·8%)	0·064	0·034	238 (6·7%)	258 (7·2%)	0·352	0·022
Unknown race^a^	420 (8·9%)	1599 (8·3%)	0·358	0·017	321 (8·9%)	307 (8·6%)	0·559	0·014
Ethnicity, n (%)								
Not hispanic or latino	2667 (74·7%)	10,525 (74·0%)	0·386	0·016	2665 (74·7%)	2617 (73·3%)	0·195	0·031
Hispanic or latino	276 (7·7%)	1143 (8·0%)	0·548	0·011	276 (7·7%)	302 (8·5%)	0·259	0·027
Unknown ethnicity	628 (17·6%)	2560 (18·0%)	0·571	0·011	628 (17·6%)	650 (18·2%)	0·497	0·016
Comorbidities, n (%)								
Essential hypertension	2142 (60·0%)	8440 (59·3%)	0·470	0·014	2140 (60·0%)	2077 (58·2%)	0·129	0·036
Heart failure	301 (8·4%)	892 (6·3%)	<0·001	0·083	299 (8·4%)	299 (8·4%)	1·000	<0·001
Ischaemic heart diseases	479 (13·4%)	1831 (12·9%)	0·387	0·016	478 (13·4%)	464 (13·0%)	0·624	0·012
Chronic kidney disease	406 (11·4%)	1554 (10·9%)	0·445	0·014	405 (11·3%)	373 (10·5%)	0·224	0·029
Cerebrovascular diseases	191 (5·3%)	909 (6·4%)	0·021	0·044	191 (5·4%)	167 (4·7%)	0·193	0·031
Overweight and obesity	960 (26·9%)	4110 (28·9%)	0·018	0·045	959 (26·9%)	927 (26·0%)	0·390	0·020
Dyslipidaemia	1877 (52·6%)	7589 (53·3%)	0·406	0·016	1876 (52·6%)	1787 (50·1%)	0·035	0·050
Medications, n (%)								
Antineoplastic hormones	14 (0·4%)	89 (0·6%)	0·100	0·033	14 (0·4%)	10 (0·3%)	0·413	0·019
Antineoplastic chemotherapy	14 (0·4%)	75 (0·5%)	0·306	0·020	14 (0·4%)	14 (0·4%)	1·000	<0·001
Trastuzumab	10 (0·3%)	11 (0·1%)	0·002	0·048	10 (0·3%)	10 (0·3%)	1·000	<0·001
Biguanides (Metformin)	1121 (31·4%)	4705 (33·1%)	0·056	0·036	1121 (31·4%)	1049 (29·4%)	0·064	0·044
Sulfonylureas	535 (15·0%)	1850 (13·0%)	0·002	0·057	534 (15·0%)	469 (13·1%)	0·027	0·052
Alpha glucosidase inhibitors	10 (0·3%)	19 (0·1%)	0·052	0·032	10 (0·3%)	10 (0·3%)	1·000	<0·001
Thiazolidinediones	101 (2·8%)	332 (2·3%)	0·086	0·031	101 (2·8%)	82 (2·3%)	0·155	0·034
DPP-4 inhibitors	269 (7·5%)	976 (6·9%)	0·158	0·026	269 (7·5%)	260 (7·3%)	0·684	0·010
GLP-1 receptor agonists	248 (6·9%)	1426 (10·0%)	<0·001	0·111	248 (6·9%)	214 (6·0%)	0·102	0·039
Insulin	27 (0·8%)	263 (1·8%)	<0·001	0·096	27 (0·8%)	21 (0·6%)	0·385	0·021
Beta blockers	1053 (29·5%)	3937 (27·7%)	0·031	0·040	1051 (29·4%)	980 (27·5%)	0·063	0·044
ACE inhibitors	837 (23·4%)	3064 (21·5%)	0·014	0·046	836 (23·4%)	771 (21·6%)	0·065	0·044
ARBs	679 (19·0%)	2720 (19·1%)	0·889	0·003	678 (19·0%)	637 (17·8%)	0·211	0·030
Laboratory								
HbA1c, %, mean ± SD	7·2 ± 1·6	6·9 ± 1·5	<0·001	0·163	7·2 ± 1·6	7·1 ± 1·6	0·487	0·023
HbA1c ≥ 7%, n (%)	1108 (31·0%)	3888 (27·3%)	<0·001	0·081	1106 (31·0%)	1014 (28·4%)	0·017	0·056
HbA1c < 7%, n (%)	1264 (35·4%)	5832 (41·0%)	<0·001	0·115	1264 (35·4%)	1193 (33·4%)	0·077	0·042

PSM, propensity score matching; SGLT2i, sodium-glucose cotransporter 2 inhibitor; SMD, standardised mean difference; SD, standard deviation; DPP-4, dipeptidyl peptidase-4; GLP-1, glucagon-like peptide-1; ACE, angiotensin-converting enzyme; ARBs, angiotensin II receptor blockers; HbA1c, haemoglobin A1c.

a Unknown Race percentage calculated so each race column sums to 100%.

**Fig. 2:**
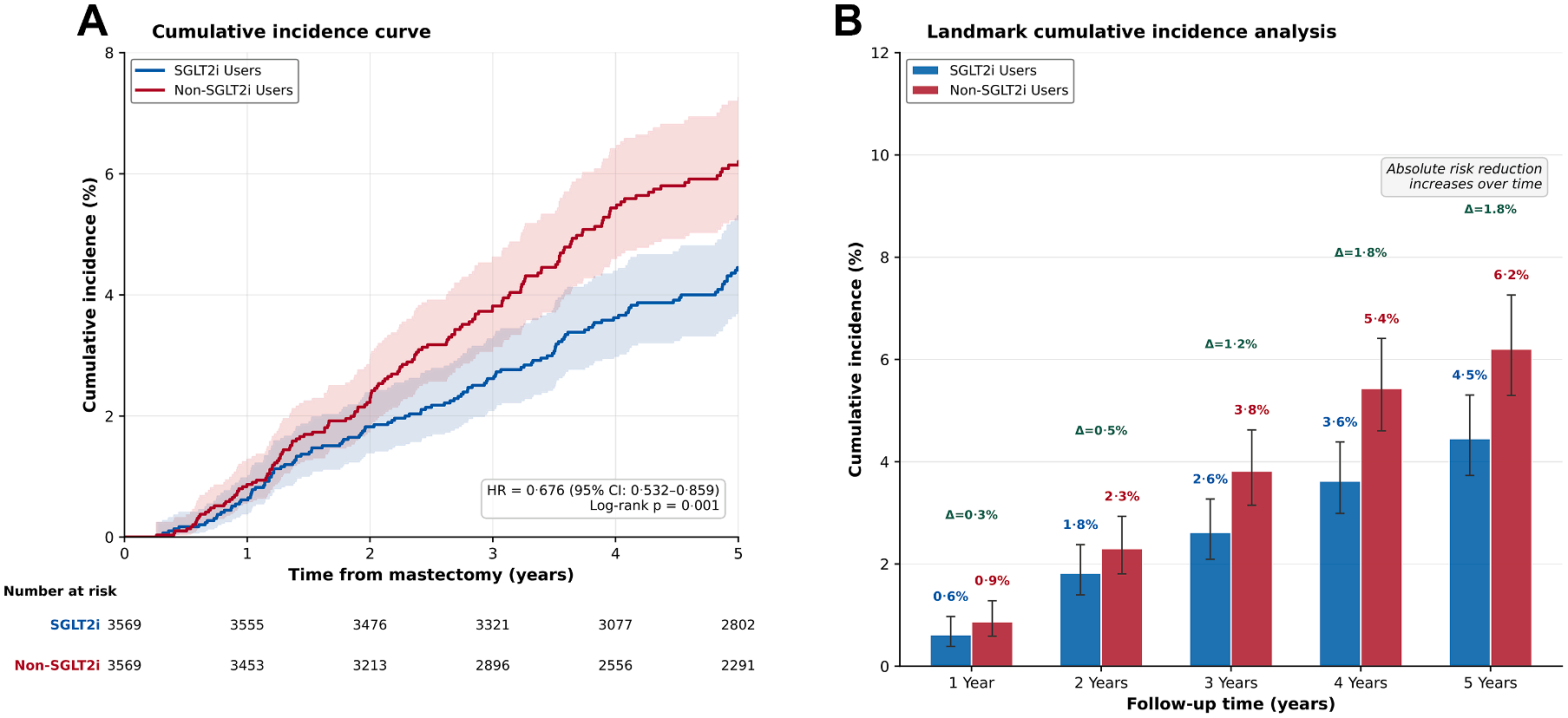
**Composite distant metastasis endpoint: SGLT2i users versus non-SGLT2i users.** (A) Cumulative incidence curves with 95% confidence bands (shaded). Follow-up begins at Day 90 post-mastectomy (landmark time point). The composite distant metastasis hazard ratio (HR) over five years was 0·676 (95% CI 0·532–0·859); log-rank p = 0·001. Numbers at risk are shown below the plot. (B) Landmark cumulative incidence analysis showing the absolute risk at one, two, three, four, and five years of follow-up. Error bars represent 95% confidence intervals around each cumulative incidence estimate. The absolute risk reduction (Δ) increased progressively from 0·3% at one year to 1·8% at four and five years. CI, confidence interval; HR, hazard ratio (the Cox proportional-hazards effect estimate used throughout; no odds ratios are reported in this figure); SGLT2i, sodium-glucose cotransporter 2 inhibitor.

At five-year follow-up, SGLT2 inhibitor use was associated with a significantly reduced risk of the composite distant metastasis endpoint compared to non-SGLT2 inhibitor use (297 events [8·3%] vs 339 events [9·5%]; HR 0·676; 95% CI 0·532–0·859; p = 0·001) (Table 2, Fig. 2A). The cumulative incidence curves began to separate after approximately two years of follow-up, with the absolute risk reduction increasing progressively over time: 0·3% at one year, 0·5% at two years, 1·2% at three years, 1·8% at four years, and 1·8% at five years (Fig. 2B).

**Table 2:** Clinical outcomes of SGLT2i users vs non-SGLT2i users.

	SGLT2i users		Non-SGLT2i users		Hazard ratio		p-value
	Numbers	Events	Numbers	Events	HR	95% CI	
Primary outcomes							
Composite metastasis endpoint^a^	3569	297	3569	339	0·676	(0·532–0·859)	0·001
Lung metastasis	3569	39	3569	61	0·550	(0·351–0·863)	<0·001
Liver metastasis	3569	29	3569	54	0·461	(0·278–0·765)	<0·001
Brain metastasis	3569	26	3569	27	0·837	(0·460–1·523)	0·481
Bone metastasis	3569	55	3569	83	0·576	(0·394–0·843)	<0·001
Bone metastasis + Skeletal-Related Events	3569	91	3569	107	0·747	(0·546–1·020)	0·068
Negative control outcomes							
Fracture (trauma)	3569	482	3569	447	0·954	(0·838–1·085)	0·475
Kidney stone	3569	243	3569	245	0·894	(0·748–1·068)	0·218

Abbreviations: SGLT2i, sodium-glucose cotransporter 2 inhibitor; HR, hazard ratio; CI, confidence interval; SRE, skeletal-related event.

a Composite metastasis endpoint = Distant Metastasis (C78 + C79).

Among organ-specific endpoints, the strongest protection was observed for liver metastasis (HR 0·461; 95% CI 0·278–0·765; p < 0·001), followed by lung metastasis (HR 0·550; 95% CI 0·351–0·863; p < 0·001) and bone metastasis (HR 0·576; 95% CI 0·394–0·843; p < 0·001) (Table 2). Brain metastasis showed a non-significant trend towards protection (HR 0·837; 95% CI 0·460–1·523; p = 0·481); given the small number of events (26 vs 27), this result is hypothesis-generating only. The composite bone metastasis plus skeletal-related events endpoint was borderline non-significant (HR 0·747; 95% CI 0·546–1·020; p = 0·068).

Time-dependent analysis of the composite distant metastasis endpoint demonstrated progressive risk separation favouring SGLT2 inhibitor users across follow-up, with the protective signal driven by sustained exposure (eTable S1, Appendix). The five-year cumulative hazard ratio was 0·676 (95% CI 0·532–0·859; p = 0·001), with a null association at three years (HR 1·007; 95% CI 0·815–1·246; p = 0·946) and an apparent early-period excess at one year (composite HR 1·587; 95% CI 1·158–2·176; p = 0·004). The early-period signal attenuated to the null under the post-hoc 180-day landmark sensitivity analysis (1-year HR 0·907; 95% CI 0·660–1·246; p = 0·546) and is interpreted as a transient artefact rather than a robust harmful effect. Parallel time-dependent patterns were observed for liver (5-year HR 0·461; 95% CI 0·278–0·765) and bone metastasis (5-year HR 0·576; 95% CI 0·394–0·843).

A post-hoc 180-day landmark sensitivity analysis, conducted to more conservatively exclude prevalent undiagnosed metastases at baseline and to test the robustness of the early-period signal, substantially eliminated the apparent excess risk observed under the primary 90-day landmark (eTable S2c, Appendix). The 1-year composite hazard ratio decreased from 1·587 (90-day landmark) to 0·907 (95% CI 0·660–1·246; p = 0·546) under the 180-day landmark. A protective signal emerged earlier at the 3-year window (HR 0·826; 95% CI 0·676–0·988; p = 0·041), and the 5-year protective association was preserved and virtually unchanged (HR 0·682; 95% CI 0·522–0·892; p = 0·005) relative to the primary 5-year estimate (HR 0·676; 95% CI 0·532–0·859). The elimination of the early-period signal under the stricter landmark, contrasted with preservation of the 5-year estimate, indicates that the late protective association is not driven by prevalent undiagnosed metastases at landmark.

The primary finding remained significant in sensitivity analyses, though with attenuated effect sizes (eTable S2, Appendix). In the post-hoc pure intention-to-treat analysis retaining comparator-cohort patients who later initiated an SGLT2 inhibitor (eTable S2b, Appendix), the hazard ratio was 0·84 (95% CI 0·76–0·93; p < 0·001). In the per-protocol analysis (patients with continuous SGLT2i exposure; N = 2358 per group), the HR was 0·82 (95% CI 0·67–0·99; p = 0·027). In the US-only population (N = 2891 per group), the HR was 0·90 (95% CI 0·81–0·98; p = 0·038). The attenuation from the ITT estimate (HR 0·676; 95% CI 0·532–0·859) towards the per-protocol (HR 0·82; 95% CI 0·67–0·99) and US-only (HR 0·90; 95% CI 0·81–0·98) estimates suggests the true effect size likely lies between these bounds.

**Fig. 3:**
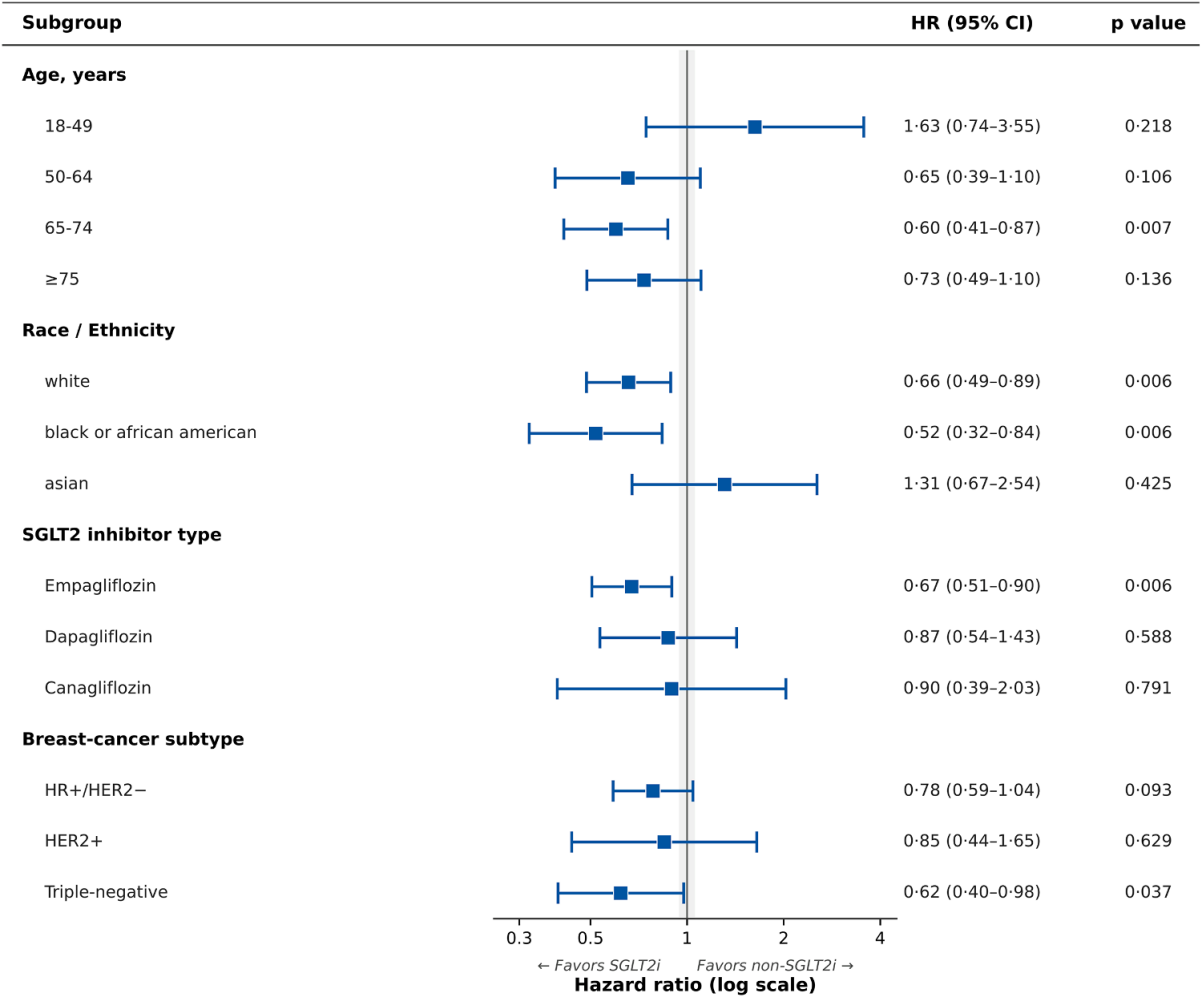
**Forest plot of subgroup analyses for the composite distant metastasis endpoint.** Hazard ratios (HR) are shown as squares with horizontal lines representing 95% confidence intervals (CI), across pre-specified subgroups including age categories, race/ethnicity, specific SGLT2 inhibitor agents, and breast cancer molecular subtype. The solid vertical line represents HR = 1·0 (no effect). P values for interaction were: age p = 0·092; race/ethnicity p = 0·066; SGLT2 inhibitor type p = 0·551; breast cancer subtype p = 0·594. All interaction tests were non-significant, supporting a class effect rather than subgroup-specific differential responses. CI, confidence interval; HR, hazard ratio; SGLT2i, sodium-glucose cotransporter 2 inhibitor; HR+/HER2−, hormone receptor-positive and human epidermal growth factor receptor 2-negative; HER2+, human epidermal growth factor receptor 2-positive.

Pre-specified subgroup analyses demonstrated consistent protective trends across patient populations (Fig. 3). No significant effect modification was observed for any subgroup: age (p-interaction = 0·092; eTable S3), race/ethnicity (p-interaction = 0·066; eTable S4), breast cancer molecular subtype (p-interaction = 0·594; eTable S5), or SGLT2 inhibitor agent (p-interaction = 0·551; eTable S6). The absence of significant interaction terms supports a class effect rather than subgroup-specific differential treatment effects.

Within individual subgroups, the strongest point estimates were observed in black or african american patients (HR 0·52; 95% CI 0·32–0·84; p = 0·006), patients aged 65–74 years (HR 0·60; 95% CI 0·41–0·87; p = 0·007), and patients with triple-negative breast cancer (HR 0·62; 95% CI 0·40–0·98; p = 0·037). Among specific agents, empagliflozin showed a significant association (HR 0·67; 95% CI 0·51–0·90; p = 0·006), while dapagliflozin (HR 0·87; 95% CI 0·54–1·43; p = 0·588) and canagliflozin (HR 0·90; 95% CI 0·39–2·03; p = 0·791) did not reach significance, likely reflecting differential statistical power rather than true heterogeneity given the non-significant p-interaction.

Traditional negative control analyses showed no significant association between SGLT2 inhibitor use and traumatic fracture (HR 0·954; 95% CI 0·838–1·085; p = 0·475) or kidney stones (HR 0·894; 95% CI 0·748–1·068; p = 0·218) (Table 2). The three post-hoc imaging-detected negative control outcomes were uniformly null across five-year follow-up: thyroid nodule (HR 1·024; 95% CI 0·836–1·255; p = 0·250), hepatic cyst (HR 0·948; 95% CI 0·745–1·205; p = 0·977), and renal cyst (HR 1·026; 95% CI 0·820–1·284; p = 0·823) (eTable S8b, Appendix), arguing against differential abdominal/thoracic imaging intensity as an explanation for the primary finding. The two post-hoc negative control exposure analyses also returned null associations for composite distant metastasis (eTable S8c, Appendix): the DPP-4 inhibitor NCE (matched N = 1328 per group; HR 1·128; 95% CI 0·863–1·473; p = 0·378) and the statin NCE (matched N = 4273 per group; HR 0·903; 95% CI 0·779–1·046; p = 0·174) were both null.

All-cause mortality, analysed as a separate endpoint within the 1:1 matched cohort at 1-, 3-, and 5-year windows (eTable S14, Appendix), did not differ significantly between groups at any window; at 5 years it was 8·81% in SGLT2 inhibitor users versus 9·24% in non-users (risk ratio 0·954, 95% CI 0·808–1·126; HR 0·986, 95% CI 0·814–1·194; log-rank p = 0·885). Because mortality was statistically indistinguishable, differential censoring by death prior to metastasis cannot meaningfully bias the cause-specific estimate. Aalen-Johansen cumulative incidence functions, computed on the unmatched eligible cohorts because the platform does not support post-match estimation, showed lower 5-year incidence in SGLT2 inhibitor users for both endpoints (distant metastasis 5·21% vs 7·63%; all-cause mortality 2·76% vs 5·69%; eFigure S2, Appendix). This unmatched analysis is descriptive cross-validation; the matched-cohort result above is the primary inference for competing risk.

E-value analysis demonstrated moderate robustness to unmeasured confounding, though potentially vulnerable to confounding by unmeasured tumour stage (eTable S7, Appendix). The E-value for the composite endpoint was 2·32 (95% CI limit: 1·60). For organ-specific outcomes, E-values were highest for liver metastasis (3·76; 95% CI limit: 1·94) and lung metastasis (3·04; 95% CI limit: 1·59), suggesting strong robustness to unmeasured confounding at these sites.

**Fig. 4:**
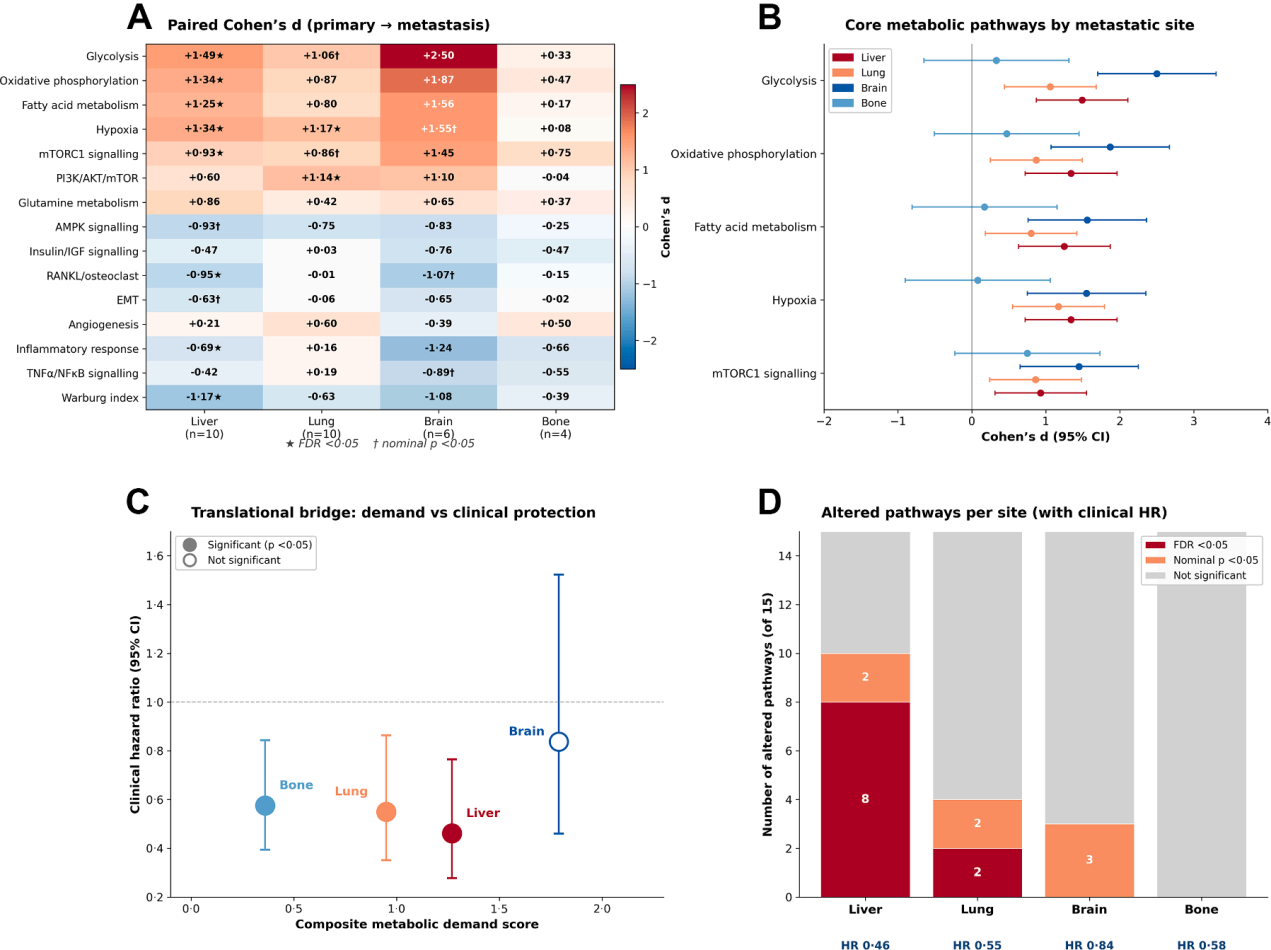
**Metabolic landscape of organ-specific breast cancer metastases supports differential SGLT2 inhibitor protection.** Data source: GSE110590 paired primary–metastasis breast cancer RNA-sequencing data (n = 16 patients, 82 samples). (A) Heatmap of paired Cohen's d values (within-patient change from primary tumour to matched metastasis) for 15 a-priori-selected pathways across four metastatic sites. ★ indicates significance at false discovery rate (FDR) < 0·05; † indicates nominal p < 0·05. (B) Forest plot of five core metabolic pathways with 95% confidence intervals by metastatic site (whiskers represent 95% CI bounds around the paired Cohen's d point estimate). (C) Translational bridge: composite metabolic demand score (x-axis, defined as the arithmetic mean of paired Cohen's d across the five core metabolic pathways) versus clinical hazard ratio (y-axis) by organ site. Filled circles indicate statistically significant clinical outcomes (p < 0·05); open circles indicate non-significant clinical outcomes. (D) Stacked bar chart showing the number of significantly altered pathways per metastatic site, annotated with clinical hazard ratios. CI, confidence interval; FDR, false discovery rate (Benjamini-Hochberg correction); Glyc-OXPHOS, the combined glycolysis and oxidative phosphorylation Cohen's d pair derived from the two Hallmark gene sets and reported jointly to capture co-ordinated energy metabolism reprogramming; HR, hazard ratio; mTORC1, mechanistic target of rapamycin complex 1; PI3K/AKT, phosphoinositide 3-kinase/protein kinase B signalling pathway; ssGSEA, single-sample gene set enrichment analysis.

In an exploratory analysis of biological plausibility, transcriptomic analysis of paired primary–metastasis specimens (GSE110590) revealed distinct metabolic reprogramming patterns across metastatic sites (Fig. 4, eTable S13, Appendix).

Liver metastases showed the broadest metabolic hyperactivity, with eight of 15 pathways significantly altered after FDR correction. Large upregulation was observed in glycolysis (Cohen's d = +1·49; FDR = 0·029), oxidative phosphorylation (d = +1·34; FDR = 0·034), fatty acid metabolism (d = +1·25; FDR = 0·034), and hypoxia (d = +1·34; FDR = 0·034). Concurrently, AMPK signalling was downregulated (d = −0·93; nominal p < 0·05) and the inflammatory response was significantly reduced (d = −0·69; FDR = 0·037).

Lung metastases demonstrated moderate metabolic upregulation, with two of 15 pathways reaching FDR significance: hypoxia (d = +1·17; FDR = 0·029) and PI3K/AKT/mTOR signalling (d = +1·14; FDR = 0·029). Brain metastases exhibited the highest individual effect sizes (glycolysis d = +2·50; oxidative phosphorylation d = +1·87), but none survived FDR correction due to limited statistical power (n = 6 pairs). Bone metastases showed no significant metabolic reprogramming (0/15 pathways at FDR<0·05).

The composite metabolic demand score—a novel exploratory descriptive metric defined as the arithmetic mean of paired Cohen's d values across five core metabolic pathways (eMethods S2, Appendix)—was highest for brain (1·79), followed by liver (1·27), lung (0·95), and bone (0·36) (Fig. 4C). Liver and lung metastases, which combine high metabolic demand with high drug exposure, showed the strongest protection. Brain metastases, despite the highest metabolic demand, showed no clinical protection, consistent with limited SGLT2 inhibitor penetration across the blood–brain barrier (brain-to-serum AUC ratio ∼0·3–0·5).^26^ Bone metastases showed clinical protection despite minimal metabolic reprogramming, suggesting an alternative protective mechanism.

## Discussion

In this large-scale target trial emulation involving 7138 propensity score-matched patients with breast cancer and type 2 diabetes, SGLT2 inhibitor use after mastectomy was associated with a 32·4% reduction in distant metastasis risk over five years. This protection was organ-specific, with the strongest effects for liver (53·9% reduction) and lung (45·0% reduction) metastasis. An exploratory transcriptomic analysis provided biological context for this gradient, showing that the sites with greatest clinical protection exhibited the most pronounced metabolic hyperactivity relative to matched primary tumours.

Our findings extend the emerging evidence for oncologic benefits of SGLT2 inhibitors. Shi and colleagues reported that SGLT2 inhibitor use was associated with delayed hormone therapy failure in prostate cancer (HR 0·63; 95% CI 0·41–0·95).12 Our composite distant metastasis HR of 0·676 (95% CI 0·532–0·859) is remarkably similar despite differences in cancer type, outcome definition, and population, and this consistency across two independent studies strengthens the plausibility of a real pharmacological effect. Our study adds the novel finding of organ-specific differential protection, not previously reported.

Rogava and colleagues demonstrated that loss of PIP4K2C confers liver-metastatic organotropism through insulin-dependent PI3K/AKT pathway activation, and that SGLT2 inhibition could reduce liver metastasis burden in a melanoma model,^11^ providing preclinical support for organ-specific protection. Our clinical findings, showing the strongest protection for liver metastasis, are directionally consistent with these preclinical observations and extend them to human breast cancer.

The organ-specific pattern suggests two complementary mechanisms (eFigure S1, Appendix). For liver and lung metastases, we propose that SGLT2 inhibitor-induced reduction in systemic glucose availability creates an energy supply–demand mismatch at metabolically hyperactive niches (Mechanism A); our transcriptomic analysis showed liver metastases exhibited comprehensive hyperactivity across glycolysis, oxidative phosphorylation, fatty acid metabolism, and hypoxia – precisely the programmes most vulnerable to glucose restriction—and first-pass hepatic exposure further amplifies this mismatch at the liver.

For bone metastasis, which showed significant clinical protection (HR 0·576; 95% CI 0·394–0·843) despite the absence of local metabolic reprogramming in transcriptomic analysis, we propose that protection operates primarily through systemic pathways (Mechanism B). SGLT2 inhibitors activate AMPK,^7^ reduce circulating insulin and IGF-1 levels,^9^ and AMPK activation has been shown to negatively regulate RANKL-induced osteoclast differentiation through suppression of oxidative stress and MAPK/NF-κB signalling,^27^^,^^28^ pathways critically implicated in bone metastasis establishment and progression.

For brain metastasis, the combination of the highest metabolic demand with limited drug delivery across the blood–brain barrier may explain the non-significant clinical finding. SGLT2 inhibitors are lipophilic and can cross the blood–brain barrier, but with brain-to-serum AUC ratios ranging from approximately 0·3 to 0·5^26^, the achieved CNS concentrations might be insufficient to meaningfully disrupt the intense metabolic demands of brain metastases. This interpretation is limited by the small clinical (26 vs 27 events) and transcriptomic (n = 6 pairs) samples and is hypothesis-generating only.

The transcriptomic analysis characterises the general metabolic landscape of metastatic tissue relative to matched primary tumours and does not directly measure SGLT2 inhibitor-specific effects; the connection between metabolic reprogramming and SGLT2 inhibitor-mediated protection is therefore inferential and hypothesis-generating.

The principal protective signal—a 32·4% reduction in composite distant metastasis at five years (HR 0·676; 95% CI 0·532–0·859; p = 0·001)—emerged progressively after approximately two years of follow-up. This time-varying pattern departs from the proportional-hazards assumption and is consistent with a biologically progressive mechanism that manifests only after sustained exposure. An apparent early-period excess at one year (composite HR 1·587; 95% CI 1·158–2·176; p = 0·004) attenuated completely under the post-hoc 180-day landmark sensitivity analysis (eTable S2c, Appendix) and is best interpreted as a transient artefact rather than a robust harmful effect. We nonetheless examine three potential non-causal mechanisms for the early signal—detection (surveillance) bias, protopathic bias, and residual immortal-time bias—each addressable through a separate empirical probe.

Detection bias from differential healthcare utilisation is the most parsimonious explanation for the early signal. SGLT2 inhibitor initiators receive monitoring for euglycaemic ketoacidosis, genitourinary infection, and renal function during the first months of therapy, and this contact intensity may surface clinically silent prevalent metastases earlier in the SGLT2i arm. The falsifiable prediction is that conditions detected through the same imaging modalities as distant metastasis (computed tomography, magnetic resonance imaging, ultrasound) but with no pharmacological link to SGLT2 inhibitors should themselves show an early excess. The three post-hoc imaging-detected negative control outcomes—thyroid nodule, hepatic cyst, and renal cyst—were uniformly null (all HR within 0·94–1·03; all p > 0·20; eTable S8b), as were the traditional negative control outcomes. This convergent null pattern argues that differential imaging intensity or healthcare-encounter frequency cannot account for the primary finding without also producing a signal in these control conditions.

Protopathic bias—in which early metabolic decompensation, weight loss, or fatigue prompts both SGLT2 inhibitor initiation and a metastasis workup—cannot be excluded within a federated claims-based platform that does not capture pre-prescription subclinical symptomatology. Residual immortal-time bias unaddressed by the 90-day landmark was tested through the post-hoc 180-day landmark analysis: the 1-year estimate moved entirely to the null while the 5-year estimate was virtually unchanged (HR 0·682; 95% CI 0·522–0·892; p = 0·005), indicating that inclusion of prevalent metastases at landmark inflates only the early-period estimate and does not generate the long-term protective signal.

We also considered whether acute metabolic perturbation following initiation—ketogenesis, transient glycaemic fluctuation, or extracellular-fluid shifts—might accelerate early micrometastatic outgrowth; however, no preclinical or clinical evidence supports such a pro-metastatic mechanism, and the complete attenuation of the early-period signal under the 180-day landmark argues against genuine biological harm during early exposure.

The convergence of two independent falsification tests—null imaging-specific negative control outcomes and complete resolution of the early-period signal under the 180-day landmark, with preservation of the 5-year estimate—supports interpreting the long-term association as the net protective signal of sustained SGLT2 inhibitor exposure rather than an artefact of differential surveillance or residual prevalent disease. We interpret the five-year mITT estimate (HR 0·676; 95% CI 0·532–0·859) as the principal causal estimand within a tripartite framework: the pure ITT analysis (HR 0·84; 95% CI 0·76–0·93), which retains comparator-arm patients who later initiated an SGLT2 inhibitor, dilutes the contrast and provides an upper bound, whereas the per-protocol analysis (HR 0·82; 95% CI 0·67–0·99), restricted to continuous exposure, approximates the maximal achievable effect. That the mITT estimate falls below both bounding analyses is consistent with the new-user comparator design, and the convergence of all three specifications in the same protective direction reinforces the robustness of the association.

Two caveats apply to the traditional negative-control programme. First, kidney stones (eTable S8, Appendix) trended numerically protective (HR 0·894; 95% CI 0·748–1·068; p = 0·218), consistent with the recognised modest reduction in urolithiasis associated with SGLT2 inhibitor use; the falsification value of this endpoint therefore rests on its wide confidence interval failing to exclude the null rather than on complete a-priori independence from the exposure. Second, the three imaging-detected negative control outcomes remain the strongest single piece of evidence against differential surveillance.

Two post-hoc negative control exposure analyses provide a complementary falsification probe for confounding at the level of drug class or chronic-medication adherence. The DPP-4 inhibitor negative control exposure (NCE-1; matched N = 1328 per group), a within-antidiabetic-class comparator, returned a null association with composite distant metastasis (HR 1·128; 95% CI 0·863–1·473; p = 0·378), excluding intra-class antidiabetic confounding. The statin negative control exposure (NCE-2; matched N = 4273 per group), a general chronic-medication comparator, was likewise null (HR 0·903; 95% CI 0·779–1·046; p = 0·174), arguing against generic healthy-adherer effects. Together with the null traditional and imaging-specific negative control outcomes, this convergent five-component negative-control programme supports drug-class specificity of the observed association rather than confounding by class-level antidiabetic effects, chronic-medication adherence, or differential surveillance.

The comparator pools all non-SGLT2 inhibitor antidiabetic agents, raising the concern that intrinsic anticancer effects of metformin or GLP-1 receptor agonists might bias the contrast against SGLT2 inhibitors. Three lines of evidence argue against this. First, baseline use of every non-SGLT2 inhibitor antidiabetic class was well balanced between matched cohorts (all SMD <0·06; Table 1), so any intrinsic effect of these agents would be approximately equally distributed and could not by itself account for the differential metastasis incidence. Second, the post-hoc DPP-4 inhibitor negative control exposure was null (HR 1·128; 95% CI 0·863–1·473; p = 0·378; eTable S8c, Appendix), falsifying the hypothesis that within-class differences among comparator agents generate signals on the scale of the primary result. Third, if metformin or GLP-1 receptor agonists do exert genuine anticancer effects, their presence in the comparator arm would bias our estimate towards the null rather than away from it, so the observed composite hazard ratio of 0·676 (95% CI 0·532–0·859) represents a conservative lower bound on the true SGLT2 inhibitor effect.

Although the point estimate for triple-negative breast cancer was HR 0·62 (95% CI 0·40–0·98; p = 0·037), the formal test for interaction across molecular subtypes was non-significant (p-interaction = 0·594), indicating no statistical evidence for effect modification by TNBC status. The numerically larger TNBC point estimate is consistent with the known glycolytic dependence of this subtype,^29^^,^^30^ but should not be interpreted as demonstrating preferential TNBC benefit; the consistent directionality of point estimates across all molecular subtypes (all <1·0) supports a class-level effect rather than a subtype-specific response, and any apparent TNBC enrichment requires prospective validation.

This study has several methodological strengths. The target trial emulation framework addresses immortal time bias and prevalent user bias through new-user design, 90-day landmark analysis, and alignment of time zero at mastectomy. The large multinational database provided adequate power for organ-specific and subgroup analyses. Propensity score matching achieved excellent covariate balance (all SMD <0·1). Null negative control outcomes support the absence of systematic bias, and E-values ≥2·32 for the composite endpoint and ≥3·04 for lung and liver metastasis indicate moderate robustness to unmeasured confounding, though potential vulnerability to confounding by unmeasured tumour stage remains.

Competing risk from non-cancer mortality was addressed through a four-layer strategy. First, all-cause mortality modelled as a separate endpoint within the matched cohort did not differ significantly at 5 years (8·81% vs 9·24%; RR 0·954, 95% CI 0·808–1·126; HR 0·986, 95% CI 0·814–1·194; p = 0·885); because any competing-risk artefact would require a differential mortality that is not observed, the cause-specific and subdistribution hazards converge under this null. Second, Aalen-Johansen cumulative incidence on the unmatched eligible cohorts showed lower 5-year incidence in SGLT2 inhibitor users for both distant metastasis (5·21% vs 7·63%) and all-cause mortality (2·76% vs 5·69%); we interpret this descriptive analysis cautiously because the federated platform does not support post-match estimation and the unmatched mortality differential reflects baseline imbalance. Third, cause-specific hazard ratios were retained as the primary estimand because the target trial emulation addresses an aetiological question that cause-specific hazards are designed to answer. Fourth, formal Fine–Gray subdistribution-hazards regression was not feasible because the platform does not expose individual-level time-to-event data. Together these layers provide robust evidence that competing risk is not an alternative explanation for the observed protection (eMethods S5, Appendix).

The generalisability of these findings reflects the geographic composition of the contributing healthcare organisations. The eligible cohort was drawn from 95 healthcare organisations distributed across both US Census Divisions (73·4% of patients) and international TriNetX sub-regions (26·6%; eTable S15), supporting applicability to a broad range of high-resource healthcare systems with established electronic-record coding and prescribing infrastructure. The composition nonetheless underrepresents low- and middle-income settings, and the federated structure precludes individual-country attribution within international sub-regions. The cohort includes black or african american (≈19%) and asian (≈7%) participants alongside a predominantly white majority, supporting some racial and ethnic representativeness; race and ethnicity here are sociocultural constructs rather than biological traits, and unmeasured socioeconomic or structural drivers that may correlate with them were not modelled and represent a residual source of confounding.

The pre-specified US-only sensitivity analysis preserved the protective direction with attenuated magnitude (HR 0·90; 95% CI 0·81–0·98; p = 0·038) relative to the modified ITT estimate (HR 0·676; 95% CI 0·532–0·859). The more homogeneous confounding environment of the US-only subpopulation makes this estimate methodologically conservative. Two non-mutually-exclusive explanations could account for the attenuation: genuine between-region heterogeneity in the true effect, or residual confounding in the more diverse global cohort that is partially relieved under US-only restriction. The available data do not allow us to distinguish these mechanisms definitively, and region-specific differences in patient acuity at initiation, concomitant therapy, or surveillance intensity cannot be excluded. Against residual confounding, we balanced 26 baseline covariates (all SMD <0·1; Table 1) and assembled a convergent battery of falsification probes—five negative control outcomes, two negative control exposures, and an E-value of 2·32 (lower 95% CI bound 1·60)—all of which were reassuring. We therefore interpret the US-only HR 0·90 as a conservative anchor and the modified ITT HR 0·676 as a global-cohort estimate that may incorporate some between-region heterogeneity, with the true effect most likely lying between these bounds and remaining significant across both, as well as across the per-protocol (HR 0·82; 95% CI 0·67–0·99) and pure ITT (HR 0·84; 95% CI 0·76–0·93) analyses.

Several limitations must be acknowledged. First, as an observational study we cannot establish causality despite the target trial emulation design. Three categories of prognostic information cannot be extracted from the federated TriNetX platform: (i) tumour stage at diagnosis, among the strongest determinants of distant metastasis and plausibly correlated with SGLT2 inhibitor prescribing through clustering with cardiovascular and renal comorbidities—although our 26-covariate model matched on these comorbidities, this captures the clustering rather than stage itself; (ii) tumour grade and histology-specific features, which cannot be formally adjusted within claims-derived covariates; and (iii) detailed chemotherapy regimens, which extend beyond the antineoplastic categories captured in the model. The composite-endpoint E-value of 2·32 (lower 95% CI bound 1·60) indicates the magnitude of unmeasured confounding that would be required to explain away the association. Second, the time-dependent reversal raises concerns about detection bias and the reliability of the five-year estimate; the true effect size likely lies between the modified ITT (HR 0·676) and per-protocol (HR 0·82) estimates. Protopathic bias from subclinical metabolic decompensation prompting both initiation and metastasis workup cannot be excluded within a platform that does not capture pre-prescription symptomatology and may contribute to the early-period excess.

Third, the brain metastasis analysis was substantially underpowered (26 vs 27 events; 95% CI 0·460–1·523), and the companion transcriptomic dataset contained only six paired brain samples (GSE110590), insufficient to survive false discovery rate correction despite the highest individual effect sizes; both clinical and transcriptomic brain findings are therefore hypothesis-generating only. Fourth, the exploratory transcriptomic analysis used a single publicly available dataset without SGLT2 inhibitor-treated samples; it characterises the metabolic landscape of metastatic tissue relative to matched primary tumours but does not directly measure SGLT2 inhibitor effects, and any mechanistic inference from it is exploratory and should not be read as establishing a link between SGLT2 inhibitor pharmacology and the observed clinical protection.

Fifth, medication adherence, daily dose, and duration of SGLT2 inhibitor exposure could not be formally assessed because the platform does not capture individual-level dispensing or refill records. As a partial proxy, the per-protocol analysis restricted to continuous exposure yielded a directionally consistent estimate (HR 0·82; 95% CI 0·67–0·99), but this does not constitute a formal dose- or duration-response evaluation, and linkage to pharmacy-claims data will be needed to characterise such components in future work. Sixth, formal Fine–Gray subdistribution-hazards regression was not feasible because the platform does not expose individual-level time-to-event data; we instead addressed competing risk through the four-layer strategy described above, which together provides evidence against competing risk as an alternative explanation.

Seventh, the apparent triple-negative breast cancer prevalence of 30·4% in our cohort substantially exceeds population norms (∼10–15%) and reflects the exclusion-based definition required when receptor-status coding is incomplete: patients without any positive HR or HER2 marker (Z17 receptor-status code or subtype-defining therapy) are assigned to TNBC by absence-of-marker logic. Our TNBC category should therefore be interpreted as HR-negative and HER2-negative by available EHR codes with incomplete molecular verification, not as a histopathologically confirmed TNBC cohort.^31^ Eighth, the comparator pool is heterogeneous by design; as detailed above, baseline class proportions were balanced after matching (Table 1) and the null DPP-4 inhibitor negative control exposure (eTable S8c) argues against within-class confounding. Ninth, post-mastectomy adjuvant therapy (chemotherapy, radiotherapy, endocrine therapy, and anti-HER2 therapy) was not included in the propensity-score model because these treatments occur after time zero, and conditioning on post-baseline variables would induce collider bias; if adjuvant treatment receipt differed systematically between matched cohorts in a metastasis-reducing direction, this could contribute to the observed association, although the curative-intent mastectomy eligibility criterion constrains adjuvant-therapy candidacy and the null negative control analyses argue against confounding of the magnitude required. Tenth, the cohort was sex-restricted by design to women with breast cancer; these findings therefore do not generalise to male breast cancer (which accounts for approximately 1% of breast cancer diagnoses), and gender identity beyond the binary biological sex variable captured in the EHR was not available.

In this target trial emulation study of 7138 propensity score-matched patients with breast cancer and type 2 diabetes, SGLT2 inhibitor use after mastectomy was associated with a significant 32·4% reduction in distant metastasis risk over five years (HR 0·676; 95% CI 0·532–0·859; p = 0·001). Protection was organ-specific, with the strongest effects for liver and lung metastasis. In absolute terms, the 5-year incidence of composite distant metastasis was approximately 1·8 percentage points lower in the SGLT2 inhibitor cohort, corresponding to a number needed to treat of roughly 56 over 5 years to prevent one distant metastasis event; this approximation should be confirmed in prospective trials. These hypothesis-generating findings warrant prospective validation in randomised controlled trials.

## Contributors

P-HC and C-HL conceptualised the study. W-CC and P-HC did data curation and formal analysis. W-CC, P-HC, H-JJ, and H-YC did the investigation. P-HC and C-HL developed the methodology. C-HL supervised the project. W-CC and P-HC did the visualisation and wrote the original draft. H-JJ, H-YC, M-HH, and C-HL reviewed and edited the manuscript. P-HC, C-HL, and H-YC accessed and verified the underlying data. All authors had full access to all the data in the study, reviewed the final version of the manuscript, and accept responsibility for the decision to submit the manuscript for publication.

## Data sharing statement

The clinical data underlying this study are available from the TriNetX platform (https://trinetx.com/) upon reasonable request and with appropriate institutional data use agreements. Deidentified participant-level data are not available owing to the federated nature of the TriNetX network, which does not permit extraction of individual-level data. The transcriptomic data are publicly available from the Gene Expression Omnibus (accession: GSE110590). Analysis code is available from the corresponding author upon reasonable request.

## Declaration of interests

We declare no competing interests.
