# Supplementary material for: SGLT2 inhibitors and risk of distant metastasis in patients with breast cancer and type 2 diabetes: a target trial emulation with biological plausibility analysis
Source: eClinicalMedicine. 2026 Jul 28;98:104117. doi: 10.1016/j.eclinm.2026.104117 (PMC13446204; doi:10.1016/j.eclinm.2026.104117)
Supplement: Supplementary file 1 [file mmc1.docx]

**SUPPLEMENTARY MATERIALS**

SGLT2 inhibitors and risk of distant metastasis in patients with breast cancer and type 2 diabetes: a target trial emulation with biological plausibility analysis

# Table of contents

STROBE Checklist – Strengthening the Reporting of Observational Studies in Epidemiology

TARGET Checklist – Transparent Reporting of Observational Studies Emulating a Target Trial

eMethods 1. Detailed study methodology (including TTE)

eMethods 2. Exploratory transcriptomic analysis for biological plausibility

eMethods 3. Molecular subtype classification algorithm

eMethods 4. Negative control analyses

eMethods 5. Competing risks analysis

eTable 1. Primary and secondary outcomes at different follow-up periods

eTable 2. Sensitivity analyses

eTable 2b. Pure intention-to-treat sensitivity analysis (expanded)

eTable 2c. 180-Day landmark sensitivity analysis

eTable 3. Subgroup analyses by age

eTable 4. Subgroup analyses by race/ethnicity

eTable 5. Subgroup analyses by breast cancer subtype

eTable 6. Subgroup analyses by SGLT2 inhibitor agent

eTable 7. E-value analysis for unmeasured confounding

eTable 8. Negative control outcomes

eTable 8b. Imaging-specific negative control outcomes

eTable 8c. Negative control exposure analyses (DPP-4 inhibitor and statin)

eTable 9. SGLT2 inhibitor drug codes

eTable 10. Outcome definitions and ICD-10 codes

eTable 11. Propensity score matching covariates

eTable 12. Gene set definitions for pathway activity analysis

eTable 13. Complete ssGSEA results: paired pathway activity changes between primary tumour and distant metastasis (GSE110590)

eTable 14. All-cause mortality in the matched cohort

eTable 15. Geographic distribution of TriNetX healthcare organisations contributing to the matched cohort

eFigure 1. Proposed dual mechanism of SGLT2 inhibitor anti-metastatic protection in breast cancer

eFigure 2. Aalen-Johansen cumulative incidence functions for distant metastasis and all-cause mortality

**STROBE Statement–Checklist of items that should be included in reports of cohort studies**

| **Item No** | **Recommendation** | **Page / Section** |
| --- | --- | --- |
| **Title and abstract** | | |
| 1(a) | Indicate the study’s design with a commonly used term in the title or the abstract | Title: “target trial emulation” |
| 1(b) | Provide in the abstract an informative and balanced summary of what was done and what was found | Summary (Background through Funding) |
| **Introduction** | | |
| 2 | Explain the scientific background and rationale for the investigation being reported | Introduction, paragraphs 1–3 |
| 3 | State specific objectives, including any prespecified hypotheses | Introduction, last paragraph |
| **Methods** | | |
| 4 | Present key elements of study design early in the paper | Methods: Study design and data source |
| 5 | Describe the setting, locations, and relevant dates, including periods of recruitment, exposure, follow-up, and data collection | Methods: Study design and data source (TriNetX, Jan 2017–Dec 2025) |
| 6(a) | Give the eligibility criteria, and the sources and methods of selection of participants. Describe methods of follow-up | Methods: Study population and eligibility criteria; Follow-up and outcome assessment |
| 6(b) | For matched studies, give matching criteria and number of exposed and unexposed | Methods: Propensity score matching; Table 1 |
| 7 | Clearly define all outcomes, exposures, predictors, potential confounders, and effect modifiers. Give diagnostic criteria, if applicable | Methods: Treatment strategies; Follow-up and outcome assessment; eTable 10–11 |
| 8* | For each variable of interest, give sources of data and details of methods of assessment (measurement) | Methods: PSM section; eTable 9–11 (ICD-10, CPT, ATC codes) |
| 9 | Describe any efforts to address potential sources of bias | Methods: TTE framework, new-user design, 90-day landmark, mITT framework with asymmetric switching, PSM, E-values, traditional and imaging negative control outcomes, negative control exposures (DPP-4 inhibitor and statin), 180-day landmark sensitivity analysis, competing-risks four-layer strategy |
| 10 | Explain how the study size was arrived at | Results: Study population (Figure 1 flow diagram); all eligible patients included |
| 11 | Explain how quantitative variables were handled in the analyses | Methods: PSM section (HbA1c categorical <7% vs ≥7%); eTable 11 |
| 12(a) | Describe all statistical methods, including those used to control for confounding | Methods: Statistical analysis; PSM |
| 12(b) | Describe any methods used to examine subgroups and interactions | Methods: Statistical analysis (prespecified subgroups, P-interaction) |
| 12(c) | Explain how missing data were addressed | Methods: PSM section (TriNetX handles missing data within platform) |
| 12(d) | If applicable, explain how loss to follow-up was addressed | Methods: Follow-up (censored at death, loss to follow-up, or 5 years) |
| 12(e) | Describe any sensitivity analyses | Methods: Statistical analysis (mITT primary; Pure ITT, per-protocol, US-only, 180-day landmark sensitivity; time-dependent) |
| **Results** | | |
| 13(a) | Report numbers of individuals at each stage of study | Results: Study population; Figure 1 |
| 13(b) | Give reasons for non-participation at each stage | Figure 1 (exclusion reasons at each step) |
| 13(c) | Consider use of a flow diagram | Figure 1 |
| 14(a) | Give characteristics of study participants and information on exposures and potential confounders | Table 1 |
| 14(b) | Indicate number of participants with missing data for each variable of interest | Table 1 (Unknown Race/Ethnicity categories reported) |
| 14(c) | Summarise follow-up time | Figure 2 (number at risk table); Results: Follow-up |
| 15* | Report numbers of outcome events or summary measures over time | Table 2; eTable 1; eTable 14 (mortality); Figure 2 |
| 16(a) | Give unadjusted estimates and, if applicable, confounder-adjusted estimates and their precision | Table 2 (HR with 95% CI after PSM) |
| 16(b) | Report category boundaries when continuous variables were categorized | eTable 11 (HbA1c <7% vs ≥7%) |
| 16(c) | If relevant, consider translating estimates of relative risk into absolute risk for a meaningful time period | Figure 2B (absolute risk reduction at 1–5 years) |
| 17 | Report other analyses done–eg analyses of subgroups and interactions, and sensitivity analyses | Results: Sensitivity analyses (eTable 2; eTable 2b Pure ITT; eTable 2c 180-day landmark); Subgroup analyses (Figure 3, eTables 3–6); E-values (eTable 7); Negative control outcomes (eTable 8 traditional; eTable 8b imaging); Negative control exposures (eTable 8c DPP-4 inhibitor and statin); Mortality (eTable 14); HCO geography (eTable 15); Discussion: Comparator heterogeneity; Heterogeneity versus residual confounding |
| **Discussion** | | |
| 18 | Summarise key results with reference to study objectives | Discussion, paragraph 1 |
| 19 | Discuss limitations of the study, taking into account sources of potential bias or imprecision | Discussion: Limitations paragraph |
| 20 | Give a cautious overall interpretation of results considering objectives, limitations, multiplicity of analyses, results from similar studies, and other relevant evidence | Discussion, paragraphs 2–5; final paragraph |
| 21 | Discuss the generalisability (external validity) of the study results | Discussion: Generalisability paragraph (95 contributing TriNetX HCOs across US Census Divisions and international regions; eTable 15); Heterogeneity versus residual confounding paragraph; Limitations (multinational TriNetX data; US-only sensitivity analysis) |
| **Other information** | | |
| 22 | Give the source of funding and the role of the funders for the present study | Funding (TSGH-D-115176); Role of the funding source |

*Reference: von Elm E, et al. The STROBE statement: guidelines for reporting observational studies. BMJ 2007;335:806–8.*

**TARGET Statement–Checklist**

*Transparent Reporting of Observational Studies Emulating a Target Trial (TARGET)*

| **Item** | **Item name** | **Checklist item** | **Location reported** |
| --- | --- | --- | --- |
| **Abstract** | | | |
| 1a |  | Identify that the study attempts to emulate a target trial. State objectives and briefly summarize the specified target trial. | Summary: Background and Methods paragraphs |
| 1b |  | Report the data sources used for emulation. | Summary: Methods (TriNetX Global Collaborative Network) |
| 1c |  | Summarize key assumptions, statistical methods, findings and conclusions. | Summary: Methods, Findings, Interpretation |
| **Introduction** | | | |
| 2 | **Background** | Describe the scientific background and the gap in knowledge. | Introduction, paragraphs 1–3 |
| 3 | **Causal question** | Summarize the causal question. | Introduction, last paragraph (two objectives) |
| 4 | **Rationale** | Describe the rationale for emulating a target trial with the available data. | Methods: Study design and data source; no prior RCT exists for this question |
| **Methods** | | | |
| 5 | **Data sources** | Cite the data sources: original purpose, type, geographic locations, setting and time-period. | Methods: Study design and data source (TriNetX, 194M patients, 171 HCOs, Jan 2017–Dec 2025) |
| **Methods – Item 6: Target trial specification** | | | |
| 6a | **Eligibility** | Describe the eligibility criteria. | Methods: Study population; eMethods 1 |
| 6b | **Treatment strategies** | Describe the treatment strategies that would be compared. | Methods: Treatment strategies; eMethods 1 |
| 6c | **Assignment** | Report that eligible individuals would be randomly assigned. | eMethods 1 (Assignment Procedures sub-section) |
| 6d | **Follow-up** | Clarify that follow-up would start at time of assignment. Specify when follow-up would end. | Methods: Follow-up and outcome assessment; eMethods 1 |
| 6e | **Outcomes** | Describe the outcomes. | Methods: Follow-up and outcome assessment; eTable 10 |
| 6f | **Causal contrasts** | Describe the causal contrasts of interest, including effect measures. | Methods: Statistical analysis (mITT primary HR; Pure ITT and per-protocol bounding sensitivity); eMethods 1 |
| 6g | **Identifying assumptions** | Describe assumptions to identify each causal estimand. | Methods: PSM section (conditional exchangeability via PSM); Discussion: Limitations (unmeasured confounders) |
| 6h | **Data analysis plan** | Describe the data analysis procedures and statistical modelling assumptions. | Methods: Statistical analysis (KM, log-rank, Cox PH, PSM) |
| **Methods – Item 7: Target trial emulation (how each was operationalized)** | | | |
| 7a | **Eligibility** | Describe how eligibility criteria were operationalized. | Methods: Study population (ICD-10: C50, E11; CPT: 19301–19307); eMethods 1 |
| 7b | **Treatment strategies** | Describe how treatment strategies were operationalized. | Methods: Treatment strategies (ATC: A10BK); eTable 9 |
| 7c | **Assignment** | Describe how assignment was operationalized. | Methods: PSM (1:1 nearest-neighbour, greedy algorithm); eTable 11 |
| 7d | **Follow-up** | Describe how follow-up was operationalized. | Methods: Follow-up (90-day landmark; censored at death/loss to f/u/5 years) |
| 7e | **Outcomes** | Describe how outcomes were operationalized. | Methods: Follow-up and outcome assessment (ICD-10 codes); eTable 10 |
| 7f | **Causal contrasts** | Describe how causal contrasts were operationalized. | Methods: Statistical analysis (Cox PH regression, HR with 95% CI) |
| 7g.i | **Assumptions** | Describe assumptions regarding baseline confounding. | Methods: PSM section (conditional exchangeability assumed after matching on 26 covariates) |
| 7g.ii | **Assumptions** | Describe how variables related to assumptions were operationalized. | Methods: PSM; eTable 11 (complete covariate definitions); E-values (eTable 7) |
| 7h.i | **Analysis** | Describe data analysis procedures and missing data handling. | Methods: Statistical analysis; TriNetX platform analytics |
| 7h.ii | **Sensitivity** | Describe additional analyses to assess sensitivity. | Methods: Statistical analysis (Pure ITT, per-protocol, US-only, 180-day landmark); eTable 2 (and 2b Pure ITT, 2c 180-day landmark); eTable 7 E-values; eTable 8 negative control outcomes (and 8b imaging, 8c exposures) |
| **Results** | | | |
| 8 | **Participant selection** | Report numbers assessed for eligibility, eligible, and assigned. Flow diagram strongly recommended. | Results: Study population; Figure 1 |
| 9 | **Baseline data** | Describe distribution of characteristics at baseline, by treatment strategy. | Table 1 (before and after PSM, with SMD) |
| 10 | **Follow-up** | Summarize length of follow-up and reasons for end of follow-up. | Figure 2A (number at risk table, 5-year follow-up) |
| 11 | **Missing data** | Describe frequency of missing data in all variables. | Table 1 (Unknown Race, Unknown Ethnicity categories) |
| 12 | **Outcomes** | Describe frequency or distribution of each outcome, by treatment strategy. | Table 2; eTable 1 |
| 13 | **Effect estimates** | Report effect estimates with precision, including absolute and relative measures. | Table 2 (HR, 95% CI); Figure 2B (absolute risk reduction) |
| 14 | **Additional analyses** | Report results of all sensitivity analyses. | eTable 2, eTable 2b (Pure ITT), eTable 2c (180-day landmark) (sensitivity); eTables 3–6 (subgroups); Figure 3; eTable 7 (E-values); eTable 8 traditional + eTable 8b imaging + eTable 8c exposures (negative controls); eTable 14 (mortality); eTable 15 (HCO geography) |
| **Discussion** | | | |
| 15 | **Interpretation** | Provide interpretation of key findings. | Discussion, paragraphs 1–4 |
| 16 | **Limitations** | Discuss limitations considering differences between target trial and emulation, and plausibility of assumptions. | Discussion: Limitations paragraph (10 items addressing unmeasured prognostic factors and stage-CV clustering, sensitivity-analysis estimand bounds, brain metastasis underpowering, transcriptomic exploratory caveats, medication adherence/dose/duration, Fine-Gray feasibility, TNBC code-based prevalence, comparator heterogeneity, post-baseline adjuvant therapy, sex-restricted scope) |
| **Other information** | | | |
| 17 | **Ethics** | Provide IRB/ethics approval and approval numbers. | Methods: Study design (TSGHIRB No.: E202516013); Ethical approval section |
| 18 | **Registration** | State whether, when and where the study protocol was registered. | Not registered (state in manuscript if required by journal) |
| 19 | **Sharing** | Provide information on data, code, and materials accessibility. | Data sharing statement |
| 20 | **Funding** | Provide funding sources and role of funders. | Funding (TSGH-D-115176); Role of the funding source |
| 21 | **COI** | State conflicts of interest and financial disclosures. | Declaration of interests |

*Reference: Cashin AG, et al. Transparent Reporting of Observational Studies Emulating a Target Trial–The TARGET Statement. JAMA 2025; DOI: 10.1001/jama.2025.13350.*

# eMethods 1. Detailed study methodology

## Study population and eligibility criteria

This retrospective cohort study utilised the TriNetX Research Network, a global federated health research network encompassing electronic health records from over 194 million patients across 171 healthcare organizations. The study period spanned January 2017 to December 2025.

### Inclusion criteria

1. Female patients aged ≥18 years at index date
2. Diagnosis of malignant neoplasm of breast (ICD-10-CM: C50)
3. History of mastectomy procedure (CPT: 19301–19307, or equivalent SNOMED codes)
4. Diagnosis of type 2 diabetes mellitus (ICD-10-CM: E11) at any time before index date
5. Use of blood glucose-lowering drugs (ATC: A10B) after diabetes diagnosis
6. **For SGLT2i cohort:** First SGLT2 inhibitor prescription (ATC: A10BK) **initiated within 90 days after mastectomy** (i.e., between Day 1 and Day 90 post-mastectomy). No SGLT2 inhibitor prescription before mastectomy (new-user requirement).
7. **For comparator cohort:** First post-mastectomy antidiabetic prescription was a non-SGLT2 inhibitor agent, **and no SGLT2 inhibitor prescription at any time from mastectomy through end of 5-year follow-up** (complete baseline exclusion of future crossovers).

### Exclusion criteria

1. Metastatic disease (ICD-10-CM: C77–C79) within 3 months before or after mastectomy
2. Personal history of breast cancer (Z85·3) excluded from initial selection
3. Outcome events occurring before index date

## Index event definition (mITT asymmetric switching handling)

Time zero was defined as the mastectomy date for both cohorts. The primary analysis used a **modified intention-to-treat (mITT)** framework with explicit, asymmetric handling of treatment switching:

- *SGLT2i cohort* (intention-to-treat side): patients were retained in their assigned cohort regardless of subsequent discontinuation or switching during follow-up.
- *Comparator cohort* (per-protocol side at baseline): patients who initiated an SGLT2 inhibitor at any point during the 5-year follow-up window were excluded **at cohort definition**, not through post-hoc censoring. This eliminates comparator crossover contamination at baseline rather than introducing immortal time bias from informative censoring.

This Hernán-style asymmetric design prevents dilution of the comparator arm by future SGLT2i users while preserving the ITT principle on the exposed side. A post-hoc pure intention-to-treat (Pure ITT) sensitivity analysis retaining comparator-cohort crossovers (eTable 2b) and a per-protocol sensitivity analysis (continuous SGLT2i exposure; eTable 2) are reported as upper- and lower-bound estimands to bracket the mITT estimate.

## Propensity score matching methodology

Propensity scores were estimated using logistic regression with 26 baseline covariates including demographics (age, sex, race, ethnicity), comorbidities (hypertension, heart failure, ischaemic heart disease, chronic kidney disease, cerebrovascular diseases, obesity, dyslipidaemia), cancer treatment history (antineoplastic chemotherapy, antineoplastic hormones, trastuzumab), diabetes medications (metformin, sulfonylureas, alpha-glucosidase inhibitors, thiazolidinediones, DPP-4 inhibitors, GLP-1 receptor agonists, insulin), cardiovascular medications (beta-blockers, ACE inhibitors, ARBs), and laboratory values (HbA1c). A 1:1 nearest-neighbor matching was performed using a greedy algorithm. Covariate balance was assessed using standardised mean differences (SMD), with SMD <0·1 indicating adequate balance. The complete covariate list is provided in eTable 11.

## E-value calculation

E-values were calculated to assess robustness to unmeasured confounding using the method of VanderWeele and Ding (2017). For protective associations (HR <1), the E-value is computed on the inverse hazard ratio. The E-value represents the minimum strength of association (on the risk-ratio scale) that an unmeasured confounder would require – with both exposure and outcome – to fully explain the observed effect, conditional on measured covariates. E-values ≥2·0 suggest moderate robustness; E-values ≥3·0 suggest strong robustness to unmeasured confounding.

## 180-day landmark sensitivity analysis

As a post-hoc sensitivity analysis to further assess the robustness of the time-dependent pattern observed in the primary 90-day landmark analysis and to more conservatively exclude prevalent undiagnosed metastases at baseline, we repeated the primary analysis using a 180-day landmark. Patients with composite distant metastasis events occurring before Day 180 post-mastectomy were excluded; follow-up began at Day 180. Hazard ratios with 95% confidence intervals were estimated at three pre-specified cumulative time windows: 1-year (D180–D365), 3-year (D180–D1,095), and 5-year (D180–D1,825), enabling direct comparison with the corresponding time windows of the primary 90-day landmark analysis. Full numerical results are reported in eTable 2c.

## Negative control analyses

A two-tier negative control framework was used to detect residual confounding and detection bias. Pre-specified negative control outcomes (NCOs) included traditional outcomes (traumatic fracture, kidney stone), with three additional imaging-specific NCOs (thyroid nodule, hepatic cyst, renal cyst) added post-hoc and selected to interrogate detection bias arising from differential abdominal/thoracic imaging. Negative control exposures (NCEs) comprised two parallel propensity-score-matched analyses substituting DPP-4 inhibitor (within-class antidiabetic control) or statin (general chronic-medication control) as the index exposure. Full design and rationale are provided in eMethods 4.

## Target trial emulation protocol specification

| Protocol Component | Target Trial | Observational Emulation |
| --- | --- | --- |
| **Eligibility Criteria** | Adult women (≥18 years) with: breast cancer (C50), completed mastectomy, type 2 diabetes mellitus, no prior SGLT2i use, no metastatic disease (C77–C79) within ±3 months of mastectomy | Same as target trial. Eligibility assessed at mastectomy date (time zero). New-user design: SGLT2i-naïve at mastectomy. SGLT2i cohort initiates within Day 1–90 after mastectomy; comparator cohort has no SGLT2i use through 5-year follow-up. |
| **Treatment Strategies** | Strategy A: Initiate SGLT2 inhibitor after mastectomy. Strategy B: Initiate non-SGLT2i antidiabetic medication after mastectomy. All other care per standard. | Treatment assignment determined by first antidiabetic prescription within 90 days after mastectomy. Comparator arm completely excludes any subsequent SGLT2i use through 5 years. |
| **Assignment Procedures** | Randomised 1:1 at mastectomy. | 1:1 propensity score matching on 26 baseline covariates measured before mastectomy. |
| **Time Zero (Index Date)** | Mastectomy procedure date. | Mastectomy procedure date. First SGLT2i or comparator prescription must occur within 90 days after mastectomy. |
| **Follow-up Period** | Starts: 90 days after mastectomy (primary landmark). Ends: outcome occurrence, death, loss to follow-up, or 5 years. | Same as target trial for primary analysis. Post-hoc 180-day landmark sensitivity analysis (eTable 2c) further excludes prevalent undiagnosed metastases. Maximum follow-up: 1,825 days. |
| **Outcomes** | Primary: composite distant metastasis (C78–C79). Secondary: organ-specific metastasis; all-cause mortality. | Primary and organ-specific outcomes per ICD-10-CM (eTable 10). All-cause mortality analysed separately (eTable 14). |
| **Causal Contrast** | Modified intention-to-treat (primary): hazard ratio comparing metastasis risk between treatment strategies, with comparator crossovers excluded at baseline. Pure ITT and per-protocol effects reported as sensitivity bounds. Cause-specific hazard ratio is the primary estimand. | Modified ITT primary; pure ITT (eTable 2b); per-protocol (eTable 2); 180-day landmark (eTable 2c). Cause-specific HR with Aalen-Johansen CIF as complementary prognostic estimator (eMethods 5; eFigure 2). |
| **Statistical Analysis** | Kaplan-Meier; log-rank; Cox proportional hazards; subgroup analyses for effect modification. | Same. 1:1 PSM (greedy nearest-neighbor); SMD <0·1; E-values (eTable 7); two-tier negative control framework (eMethods 4). |

## Target trial emulation study design schematic

**Study Design Timeline (90-day initiation window; mITT primary analysis)**

|  | BASELINE | TIME ZERO | INITIATION WINDOW | LANDMARK | FOLLOW-UP |
| --- | --- | --- | --- | --- | --- |
| Period | Pre-mastectomy | Mastectomy | Day 1–90 | Day 90 | Day 90 → 5 years |
| Eligibility | Breast cancer; T2DM; SGLT2i-naïve; no metastasis | Mastectomy procedure; index date set | First antidiabetic prescription must fall here | Must survive to Day 90 with no event | Outcome ascertainment; censored at death, loss to f/u, or 5 years |
| Treatment | No SGLT2i use (required) | – | SGLT2i cohort: A10BK initiated. Comparator cohort: non-SGLT2i antidiabetic initiated, AND no future SGLT2i through 5y | Treatment continues (mITT: as assigned for SGLT2i arm; baseline-excluded for comparator crossovers) |  |
| Covariates | 27 PSM covariates measured here | – | – | – | – |

### Key design features

| Feature | Implementation |
| --- | --- |
| New-user, restricted-window design | Patients must have no SGLT2i prescriptions before mastectomy and (for the SGLT2i cohort) initiate within 90 days after. This captures treatment initiation, anchors time zero, and avoids prevalent user bias. |
| Comparator integrity | Patients with any SGLT2i prescription during follow-up were excluded at baseline from the comparator cohort. This prevents post-baseline contamination without introducing informative censoring. |
| Time zero alignment | Mastectomy date serves as time zero for both cohorts, aligning eligibility, treatment assignment, and follow-up start. This prevents immortal time bias from misaligned origins. |
| 90-day primary landmark | Outcome ascertainment begins at Day 90, providing a treatment-initiation window. Post-hoc 180-day landmark sensitivity analysis (eTable 2c) further excludes prevalent undiagnosed metastases. |
| Baseline covariate timing | All PSM covariates measured before mastectomy (time zero) so they are not affected by treatment assignment. |
| Modified ITT analysis | SGLT2i arm analysed per intention-to-treat; comparator arm constructed at baseline to exclude future SGLT2i users. Pure ITT (eTable 2b) and per-protocol (eTable 2) provided as sensitivity bounds. |

### Biases addressed by this design

| Bias Type | How Addressed |
| --- | --- |
| Immortal time bias | 90-day landmark (and 180-day sensitivity); time zero anchored at mastectomy for both groups; comparator crossovers excluded at baseline (not censored). |
| Prevalent user bias | New-user design excludes patients with SGLT2i exposure before mastectomy. |
| Selection bias | 1:1 PSM on 26 baseline covariates; exclusion of prevalent users prevents depletion of susceptibles. |
| Confounding by indication | PSM on diabetes severity (HbA1c), comorbidities, prior medications, cancer treatments. |
| Comparator contamination dilution | mITT asymmetric framework: comparator cohort excludes all future SGLT2i users at baseline. |
| Unmeasured confounding | E-value analysis (eTable 7); negative control outcomes (eTables 8, 8b); negative control exposures (eTable 8c). |
| Detection bias | 180-day landmark sensitivity (eTable 2c); imaging-specific NCOs (eTable 8b). |
| Competing risks | All-cause mortality endpoint (eTable 14); Aalen-Johansen CIF (eFigure 2); cause-specific HR estimand justification (eMethods 5). |

# eMethods 2. Exploratory transcriptomic analysis for biological plausibility

## Rationale

To explore potential biological mechanisms underlying the organ-specific pattern of metastasis protection observed in the clinical cohort, we performed an exploratory transcriptomic analysis using publicly available paired primary–metastasis RNA-sequencing data. This analysis aimed to characterise the metabolic landscape of breast cancer distant metastases at different organ sites and to assess whether observed metabolic reprogramming patterns were directionally consistent with the known pharmacological properties of SGLT2 inhibitors. This analysis provides biological context rather than direct validation, as the transcriptomic dataset does not contain SGLT2 inhibitor–treated samples.

## Data source

We utilised the GSE110590 dataset (Siegel MB, He X, Hoadley KA, Hoyle A, Pearce JB, Garrett AL, et al. Integrated RNA and DNA sequencing reveals early drivers of metastatic breast cancer. *J Clin Invest.* 2018;128(4):1371–1383. doi:10.1172/JCI96153), a paired primary–metastasis RNA-sequencing dataset generated on the Illumina HiSeq platform. The dataset contains log2-normalised expression data for 15,389 genes across 82 samples from 16 patients with breast cancer. Each patient contributed paired primary tumour and distant metastasis samples, enabling within-patient comparisons that control for inter-individual genomic heterogeneity. The paired sample distribution across metastatic sites was: liver (n = 10 pairs), lung (n = 10 pairs), brain (n = 6 pairs), and bone (n = 4 pairs). Because some patients contributed metastasis samples from multiple organ sites, the total number of paired comparisons exceeds the number of patients.

## Data processing

Gene identifiers in the original dataset were formatted as Symbol|EntrezID. We parsed EntrezID values and mapped them to HUGO gene symbols using the *org.Hs.eg.db* annotation database (Bioconductor). Samples were classified by metastatic site based on sample title annotation in the GEO metadata; ambiguous annotations (e.g., RIB, SPINAL) were manually mapped to the appropriate organ site (bone) by reviewing the original clinical descriptions. Paired sample assignments were verified by confirming that each primary–metastasis pair originated from the same patient identifier.

## Gene set selection

We curated 14 biologically relevant gene sets and one composite index (WARBURG_INDEX) for pathway activity analysis. Gene sets were selected *a priori* based on two criteria: (1) pathways directly relevant to glucose and energy metabolism, representing the pharmacological targets of SGLT2 inhibitors (glycolysis, oxidative phosphorylation, fatty acid metabolism, hypoxia, mTORC1 signalling, PI3K/AKT/mTOR, glutamine metabolism, AMPK signalling, insulin/IGF signalling); and (2) pathways relevant to metastatic microenvironment biology (RANKL/osteoclast signalling, epithelial–mesenchymal transition, angiogenesis, inflammatory response, TNFα/NFκB signalling). Ten gene sets were derived from the Molecular Signatures Database Hallmark collection (MSigDB v2023·2, Broad Institute), and four were custom-assembled from published literature. Complete gene lists for all 14 gene sets are provided in eTable 12.

## Single-sample gene set enrichment analysis

Pathway activity scores were computed using single-sample gene set enrichment analysis (ssGSEA) as implemented in the GSVA R package (version 1·46·0; Hänzelmann et al., *BMC Bioinformatics* 2013;14:7). For each sample, ssGSEA ranks all genes by their expression level and calculates an enrichment score based on the empirical cumulative distribution functions of genes within and outside each gene set. This produces a continuous pathway activity score per sample per gene set, enabling quantitative paired comparison between primary tumour and metastatic tissue from the same patient.

The WARBURG_INDEX was computed as the difference between the glycolysis and oxidative phosphorylation ssGSEA scores (Glycolysis – OXPHOS) for each sample. Positive values indicate relative glycolytic preference (Warburg effect); negative values indicate oxidative phosphorylation dominance.

## Statistical analysis

Within-patient changes in pathway activity between primary tumour and paired metastasis were assessed using two-sided Wilcoxon signed-rank tests, performed separately for each metastatic site. This nonparametric approach was chosen given the small sample sizes at each site (n = 4 to 10 pairs).

Effect sizes were quantified using Cohen’s *d* for paired samples, calculated as *d* = M_diff_ / SD_diff_, where M_diff_ is the mean of paired differences and SD_diff_ is the standard deviation of paired differences. Effect sizes were interpreted as small (≤0·5), medium (0·5–0·8), and large (≥0·8) following standard benchmarks. Approximate 95% confidence intervals for Cohen’s *d* were computed using the normal approximation method (*d* ± 1·96/√n). Multiple testing correction was applied within each organ site using the Benjamini-Hochberg procedure to control the false discovery rate (FDR) across the 15 pathway comparisons tested per site. Results are reported at two significance thresholds: FDR <0·05 (denoted ★) and nominal P <0·05 (denoted †).

## Composite metabolic demand score

To provide a summary measure of metabolic reprogramming intensity at each metastatic site, we calculated a composite metabolic demand score defined as the arithmetic mean of paired Cohen's d values across five core metabolic pathways: glycolysis, oxidative phosphorylation, fatty acid metabolism, hypoxia, and mTORC1 signalling. These five pathways were selected a priori on the following basis: each represents a quantitative readout of tumour-cell energy phenotype with direct mechanistic relevance to the pharmacological targets of SGLT2 inhibitors (glucose flux, ATP supply, and hypoxic-stress response), and each has established gene-set definitions in MSigDB Hallmark with sufficient signal-to-noise ratio in the GSE110590 dataset. The remaining nine of the 14 gene sets in eMethods 2 (PI3K/AKT/mTOR, glutamine metabolism, AMPK signalling, insulin/IGF signalling, RANKL/osteoclast signalling, epithelial–mesenchymal transition, angiogenesis, inflammatory response, TNFα/NFκB signalling) were retained as supplementary context but were excluded from the composite to keep the score focused on glucose-energy metabolism rather than diluting it across mixed downstream and microenvironment pathways. The arithmetic mean was chosen over alternative aggregations (e.g., principal component, weighted geometric mean) to preserve interpretability on the original Cohen's d scale and to avoid post-hoc weighting that could be perceived as overfitting given the limited sample size (n=4–10 paired samples per metastatic site); under an a-priori-symmetry assumption, each of the five pathways is treated as equally informative. We emphasise that the composite metabolic demand score is a novel descriptive metric. It has not been externally validated, its clinical interpretability is constrained by the exploratory transcriptomic dataset on which it is based (GSE110590, n=16 paired samples), and it is used strictly for hypothesis-generating cross-site comparison (Figure 4, Panel C). Inferences from the score should not be extended beyond descriptive correspondence with the organ-specific clinical hazard ratios, and external validation in independent transcriptomic datasets and in SGLT2 inhibitor–treated experimental systems is required before any mechanistic interpretation.

## Methodological limitations

This exploratory analysis has several inherent methodological limitations. First, GSE110590 characterises the general metabolic features of metastatic tissue relative to matched primary tumours; it does not measure SGLT2 inhibitor–specific transcriptomic effects, and no publicly available dataset with SGLT2 inhibitor–treated cancer cell transcriptomes currently exists. Second, the link between metastatic metabolic reprogramming and SGLT2 inhibitor–mediated protection is inferential. Third, statistical power was limited for brain (n = 6 pairs) and bone (n = 4 pairs). Fourth, this is a single dataset with no independent replication possible at present. These limitations are discussed in the main text.

## Software

All transcriptomic analyses were performed in R (version 4.3.1) using the following packages: GSVA version 1·46·0 (ssGSEA computation), org.Hs.eg.db (gene identifier mapping), stats (Wilcoxon signed-rank tests, Benjamini-Hochberg correction), and effsize (Cohen’s *d*). Figure 4 was generated using Python (matplotlib, seaborn). Analysis scripts and gene set definitions are available from the corresponding author upon reasonable request.

# eMethods 3. Molecular subtype classification algorithm

## Rationale

Because TriNetX does not systematically capture genomic or immunohistochemistry assay results across contributing healthcare organisations, molecular subtype was inferred for each patient using a pre-specified hierarchical algorithm combining ICD-10-CM receptor status codes (Z17·0–Z17·421) with post-mastectomy breast-cancer-directed medication use. This treatment-pattern approach is consistent with validated claims-based algorithms for breast-cancer subtype identification (Beachler et al. Pharmacoepidemiol Drug Saf 2019).

## Hierarchical 3-step algorithm

The classifier was applied in the following order. Each patient was classified into the first matching category, with later steps applied only to patients not classified in earlier steps:

**Step 1 – HER2-positive (any HR status)**

Inclusion (any of):

- ICD-10-CM receptor status: Z17·31 (oestrogen receptor positive, HER2 receptor positive), or 410, or 420 (alternative HER2-positive coding patterns)
- Any post-mastectomy anti-HER2 medication use (within 5-year follow-up window):
  - Trastuzumab (intravenous or subcutaneous; trade names Herceptin, Herceptin Hylecta)
  - Pertuzumab (Perjeta)
  - Trastuzumab-emtansine / T-DM1 (Kadcyla)
  - Trastuzumab-deruxtecan / T-DXd (Enhertu) – see Special Cases below
  - Lapatinib (Tykerb)
  - Neratinib (Nerlynx)
  - Tucatinib (Tukysa)
  - Margetuximab (Margenza)

**Step 2 – HR-positive / HER2-negative** (applied only to patients NOT classified as HER2-positive in Step 1)

Inclusion (any of):

- ICD-10-CM receptor status: Z17·0 (oestrogen receptor positive), or Z17·21 (oestrogen receptor negative, progesterone receptor positive – treated as HR-positive), or Z17·41, or Z17·411 (alternative HR-positive coding)
- Any post-mastectomy endocrine therapy or CDK4/6 inhibitor use (within 5-year follow-up window):
  - Selective oestrogen receptor modulators: tamoxifen, toremifene
  - Selective oestrogen receptor degraders: fulvestrant, elacestrant
  - Aromatase inhibitors: anastrozole, letrozole, exemestane
  - GnRH agonists used for ovarian suppression: leuprolide, goserelin (when paired with another endocrine agent)
  - CDK4/6 inhibitors: palbociclib, ribociclib, abemaciclib
  - mTOR inhibitor (in HR+ context): everolimus
  - PI3K inhibitor (in HR+ context): alpelisib

**Step 3 – Triple-negative (TNBC), by exclusion**

All remaining patients were classified as triple-negative breast cancer.

## Complete ICD-10-CM receptor status codes referenced

| Code | Definition |
| --- | --- |
| Z17·0 | Oestrogen receptor positive status (ER+) |
| Z17·21 | Oestrogen receptor negative, progesterone receptor positive |
| Z17·31 | Oestrogen receptor positive, HER2 receptor positive |
| Z17·41 | HER2 receptor positive status |
| Z17·411 | HER2 receptor positive status (alternative subcoding) |
| Z17·4– | HER2-related coding family (including timing-relevant subcodes) |
| 410, 420 | Alternative HER2-positive coding patterns observed in claims data |

## Special cases and edge conditions

- **Trastuzumab-deruxtecan (T-DXd / Enhertu):** This agent is indicated for HER2-low (IHC 1+, IHC 2+/ISH−) as well as HER2-positive disease. For algorithmic consistency, T-DXd use was treated as evidence of HER2-positive status when HER2 IHC 0 was not separately documented; this represents a small population in our cohort.
- **Z17·4– timing:** Only HER2-related Z-codes recorded at or before the post-mastectomy index window were used to define HER2-positive status; codes documented after a metastasis event were not used to retro-classify the primary tumour.
- **Concurrent chemotherapy:** Use of chemotherapy alone (e.g., anthracycline–taxane regimens) was not used as a subtype-defining criterion, as these agents are administered across all subtypes.

## Acknowledged limitations

This algorithmic classification is subject to four documented limitations:

1. **Reliance on treatment patterns.** Subtype assignment depends on observed medication exposure post-mastectomy. A patient who would have received endocrine or anti-HER2 therapy but did not (e.g., due to early death, low compliance, or contraindication) may be misclassified into a more inclusive (e.g., TNBC) category by exclusion.
2. **Higher-than-expected TNBC proportion.** The observed TNBC proportion in our cohort (30·4%) exceeds population prevalence (commonly reported as 10–20%). This is an expected mechanical consequence of the exclusion-based definition when receptor status coding is incomplete, as patients without documented receptor codes or definitive subtype-specific therapies fall to the TNBC category by default.
3. **Non-differential misclassification.** Misclassification, if present, is non-differential between SGLT2 inhibitor and comparator arms, as receptor status coding and subtype-defining therapy patterns do not depend on antidiabetic prescription. Non-differential misclassification of an effect modifier biases interaction tests toward the null. The non-significant subtype interaction (p-interaction = 0·594; eTable 5) should therefore be interpreted in this light: a true subtype-level differential effect, if present, may have been attenuated by misclassification.
4. **Z17 coding completeness.** Z17 codes are administrative supplementary codes whose recording rate varies across TriNetX healthcare organisations. The treatment-pattern arm of the algorithm (Steps 1–2 medication criteria) was added specifically to mitigate this limitation.

## TriNetX implementation notes

This algorithm was operationalised on the TriNetX platform using sequential cohort filters (Z-code receptor status combined with post-index medication exposure). Patient counts at each step were tracked: HER2-positive (Step 1) → 6·4% of matched cohort; HR+/HER2− (Step 2) → 63·2%; TNBC (Step 3) → 30·4%. Subgroup hazard ratios are reported in eTable 5 with the recommendation that the TNBC subgroup point estimate (HR 0·62) be interpreted in the context of the non-significant subtype interaction test and the documented misclassification considerations above.

# eMethods 4. Negative control analyses

We conducted two complementary sets of negative control analyses to address residual confounding and detection bias.

## 4·1 Negative control outcomes (NCOs)

**Traditional NCOs.** Traumatic fracture (ICD-10-CM S02–S92, excluding pathological fractures) and kidney stone (N20–N23) were pre-specified as outcomes unrelated to breast cancer biology and unrelated to SGLT2 inhibitor pharmacology (Lipsitch M et al., *Epidemiology* 2010). Significant associations would suggest unmeasured confounding or systematic bias in healthcare utilisation.

**Imaging-specific NCOs.** To specifically test imaging-driven detection bias, three additional NCOs were added as post-hoc analyses. These are conditions incidentally detected through the same abdominal and thoracic imaging modalities (CT, MRI, ultrasound) used to diagnose distant metastasis, but without biological relationship to SGLT2 inhibitor pharmacology:

- **Thyroid nodule** (ICD-10-CM E04.1) – incidental finding on chest CT performed during pulmonary metastasis evaluation
- **Hepatic cyst** (ICD-10-CM K76.89) – incidental finding on abdominal CT/MRI performed during hepatic metastasis evaluation
- **Renal cyst** (ICD-10-CM N28.1) – incidental finding on abdominal CT/MRI performed during the same imaging studies

Conditions with plausible SGLT2 inhibitor pharmacological links (e.g., non-alcoholic fatty liver disease, complex renal lesions) were excluded *a priori* from NCO selection. If differential imaging utilisation between cohorts were biasing the primary analysis, we would expect to see correspondingly different rates of these incidentally detected conditions; null associations across all three would argue against this bias mechanism.

## 4·2 Negative control exposures (NCEs)

Two post-hoc parallel 1:1 propensity-score-matched negative control exposure analyses (to falsify class-level antidiabetic and chronic-medication adherence confounding mechanisms) were conducted using the same target trial emulation framework as the primary analysis, with the index exposure substituted; cohort eligibility was redefined around the substituted index drug, and matched cohort sizes therefore differ from the primary SGLT2 inhibitor analysis:

**NCE-1 – Class-level antidiabetic control (DPP-4 inhibitor versus non-DPP-4 inhibitor).**

This analysis tests whether the observed SGLT2 inhibitor association reflects SGLT2 inhibitor–specific pharmacology or within-antidiabetic-class confounding shared with other oral antidiabetic agents. Cohort 1: DPP-4 inhibitor users (ATC A10BH); Cohort 2: non-DPP-4 inhibitor users on other antidiabetic therapy. Both arms excluded any SGLT2 inhibitor use to avoid contamination of the negative-control comparison.

**NCE-2 – General chronic-medication control (statin versus non-statin).**

This analysis tests whether the observed SGLT2 inhibitor association reflects general features of chronic medication use (adherence-related healthy-user effects, regular contact with care) rather than drug-specific pharmacology. Cohort 1: statin users (ATC C10AA); Cohort 2: non-statin users, drawn from the same diabetic post-mastectomy eligibility population. Both arms excluded any SGLT2 inhibitor use.

**Common methodology.** Each NCE analysis applied the same baseline covariate framework as the primary analysis (eTable 11). For the DPP-4 inhibitor negative control exposure, the DPP-4 inhibitor variable was removed from the diabetes-medication covariate group to avoid collinearity with the index exposure. For the statin negative control exposure, statin is not included in the primary covariate set; dyslipidaemia (a covariate already present) partially captures statin indication. Outcomes, follow-up structure, and analytic models were identical to the primary analysis. Results are reported in eTable 8c.

# eMethods 5. Competing risks analysis

To address potential competing risk from non-cancer mortality in this older diabetic population, we performed a post-hoc four-layer evidence strategy.

## 5·1 All-cause mortality as a separate endpoint

Using the same 1:1 propensity-score-matched cohort, we analysed all-cause mortality at 1-year, 3-year, and 5-year follow-up windows. Risk ratios and hazard ratios were estimated using the same analytic framework as the primary metastasis analysis. Results are presented in eTable 14.

## 5·2 Aalen-Johansen cumulative incidence function estimation

We conducted a formal competing risks analysis using the Aalen-Johansen non-parametric estimator to compute cumulative incidence functions (CIFs) for both distant metastasis and all-cause mortality, treating each as a competing event for the other. The Aalen-Johansen estimator is the non-parametric gold standard for competing risk analysis; it does not require the proportional subdistribution hazards assumption of Fine-Gray models and is therefore methodologically robust without imposing parametric assumptions. CIF curves with 95% confidence intervals are presented in eFigure 2. These Aalen-Johansen analyses were conducted on the unmatched eligible cohorts because the federated TriNetX platform supports cumulative-incidence-function estimation only at the cohort level and does not support post-match estimation; the resulting CIF estimates are therefore presented as descriptive cross-validation, not as primary effect estimates.

## 5·3 Estimand justification

Cause-specific hazard ratios remained the primary estimand because our target trial emulation addresses an aetiological research question: *“Does SGLT2 inhibitor use change the biological rate of distant metastasis?”* This is the question cause-specific hazards are designed to answer (Young JG et al., *Stat Med* 2020; Andersen PK et al., *Int J Epidemiol* 2012; Latouche A et al., *J Clin Epidemiol* 2013). Subdistribution (Fine-Gray) hazard ratios are appropriate when the question of interest is the absolute probability of the event of interest in the presence of the competing event – a prognostic, not aetiological, question. The Aalen-Johansen CIFs presented here provide the complementary prognostic perspective regarding absolute event probabilities.

## 5·4 Platform constraint

Formal Fine-Gray subdistribution hazard regression was not feasible within the federated TriNetX platform, which does not support extraction of individual-level time-to-event data required for subdistribution hazard estimation with covariate adjustment. The combination of the separate mortality endpoint analysis, Aalen-Johansen CIFs, the qualitative pattern of simultaneous reduction in both events under SGLT2i exposure, and the cause-specific estimand justification provides a comprehensive response to competing-risk concerns without requiring individual-level modelling.

# eTable 1. Primary and secondary outcomes at different follow-up periods

N = 3,569 per group after propensity score matching. Modified ITT analysis with 90-day landmark.

| Outcome | Follow-up | SGLT2i Events | Non-SGLT2i Events | HR (95% CI) | P-value |
| --- | --- | --- | --- | --- | --- |
| Composite Metastasis* | 5 years | 297 | 339 | 0·676 (0·532–0·859) | 0·001 |
|  | 3 years | 154 | 192 | 1·007 (0·815–1·246) | 0·946 |
|  | 1 year | 93 | 66 | 1·587 (1·158–2·176) | 0·004 |
| Lung Metastasis | 5 years | 49 | 61 | 0·550 (0·351–0·863) | <0·001 |
|  | 3 years | 39 | 58 | 1·065 (0·727–1·558) | 0·747 |
|  | 1 year | 28 | 18 | 1·757 (0·972–3·177) | 0·059 |
| Liver Metastasis | 5 years | 42 | 54 | 0·461 (0·278–0·765) | <0·001 |
|  | 3 years | 29 | 38 | 1·399 (0·901–2·171) | 0·133 |
|  | 1 year | 25 | 13 | 2·162 (1·106–4·227) | 0·021 |
| Brain Metastasis | 5 years | 26 | 27 | 0·837 (0·460–1·523) | 0·481 |
|  | 3 years | 23 | 27 | 1·109 (0·635–1·936) | 0·716 |
|  | 1 year | N/A | N/A | N/A | N/A |
| Bone Metastasis | 5 years | 79 | 83 | 0·576 (0·394–0·843) | <0·001 |
|  | 3 years | 66 | 55 | 1·513 (1·091–2·100) | 0·013 |
|  | 1 year | 46 | 18 | 2·847 (1·651–4·911) | <0·001 |
| Bone Metastasis + SREs | 5 years | 91 | 107 | 0·747 (0·546–1·020) | 0·068 |
|  | 3 years | 71 | 64 | 1·114 (0·978–1·269) | 0·105 |
|  | 1 year | 43 | 32 | 1·216 (1·032–1·434) | 0·019 |

*Composite Metastasis Endpoint = Distant Metastasis (C78 + C79). Bold values indicate statistical significance (P < 0·05). SREs = Skeletal-Related Events.*

# eTable 2. Sensitivity analyses

Sensitivity analyses for primary outcome (Composite Metastasis Endpoint).

| Analysis | N per group | HR (95% CI) | P-value |
| --- | --- | --- | --- |
| Main Analysis (mITT, 90-day landmark, 5-year) | 3,569 | 0·68 (0·53–0·86) | 0·001 |
| Pure ITT (comparator crossovers retained) | 3,486 | 0·84 (0·76–0·93) | <0·001 |
| Per-Protocol (continuous SGLT2i exposure) | 2,358 | 0·82 (0·67–0·99) | 0·027 |
| 180-day landmark, 5-year | 3,569 | 0·682 (0·522–0·892) | 0·005 |
| US-Only Population, 5-year | 2,891 | 0·90 (0·81–0·98) | 0·038 |
| 3-Year Follow-up (90-day landmark) | 3,569 | 1·01 (0·82–1·25) | 0·946 |
| 1-Year Follow-up (90-day landmark) | 3,569 | 1·59 (1·16–2·18) | 0·004 |
| 3-Year Follow-up (180-day landmark) | 3,569 | 0·826 (0·676–0·988) | 0·041 |
| 1-Year Follow-up (180-day landmark) | 3,569 | 0·907 (0·660–1·246) | 0·546 |

*mITT = modified intention-to-treat. Pure ITT retains comparator-cohort patients who later initiated SGLT2 inhibitor; per-protocol restricts to patients with continuous SGLT2 inhibitor exposure throughout follow-up. The 180-day landmark sensitivity analysis excludes outcome events occurring before Day 180 post-mastectomy.*

# eTable 2b. Pure intention-to-treat sensitivity analysis (expanded)

A post-hoc sensitivity analysis in which all patients in the comparator cohort were retained regardless of whether they later initiated an SGLT2 inhibitor during follow-up. All other study procedures (eligibility, propensity score covariates, outcome definition, follow-up structure) were identical to the primary analysis.

| Parameter | SGLT2i (Cohort 1) | Non-SGLT2i (Cohort 2, Pure ITT) |
| --- | --- | --- |
| Pre-match N | 3,591 | 17,691 |
| Post-match N | 3,486 | 3,486 |
| Composite metastasis events (5-year) | 209 (6·0%) | 280 (8·0%) |
| Mean follow-up (days, post-match) | 883 | 894 |
| Median follow-up (days, post-match) | 788 | 771 |
| 5-year Kaplan-Meier survival (metastasis-free) | 90·35% | 89·24% |
| Effect Measure | Value (95% CI) | P-value |
| Risk Difference (5-year) | −2·0% (−3·2% to −0·8%) | 0·001 |
| Risk Ratio (5-year) | 0·75 (0·63–0·89) | 0·001 |
| Odds Ratio (5-year) | 0·73 (0·61–0·88) | <0·001 |
| Cox Hazard Ratio | **0·84 (0·76–0·93)** | <0·001 |
| Log-rank χ² | 10·34 | 0·001 |
| Proportional hazards assumption (test) | – | p = 0·287 (assumption met) |

*Interpretation: The Pure ITT estimate (HR 0·84) is attenuated relative to the modified ITT primary analysis (HR 0·68), as expected from inclusion of comparator-cohort patients who subsequently initiated SGLT2 inhibitors. The estimate remains protective and statistically significant.*

# eTable 2c. 180-day landmark sensitivity analysis

A post-hoc sensitivity analysis with extended landmark (180 days post-mastectomy) to more conservatively exclude prevalent undiagnosed metastases at baseline. Patients with composite distant metastasis events occurring before Day 180 post-mastectomy were excluded; follow-up began at Day 180.

| Follow-up window | N per group | Composite distant metastasis HR (95% CI) | P-value | Comparison vs 90-day landmark |
| --- | --- | --- | --- | --- |
| 1-year (D180–D365) | 3,569 | **0·907 (0·660–1·246)** | 0·546 | Resolves apparent harm seen at 1-year 90-day landmark (HR 1·587) |
| 3-year (D180–D1,095) | 3,569 | **0·826 (0·676–0·988)** | **0·041** | Earlier emergence of protective signal compared with 90-day landmark (HR 1·01 at 3-year) |
| 5-year (D180–D1,825) | 3,569 | **0·682 (0·522–0·892)** | 0·005 | Virtually identical to 90-day primary estimate (HR 0·676) |

*The substantial attenuation of the 1-year hazard ratio (1·587 → 0·907) when the landmark is extended from 90 to 180 days is consistent with detection of prevalent undiagnosed metastases unmasked by SGLT2 inhibitor–related care intensification in the early post-mastectomy period. The 5-year estimate is robust to this design variation, indicating that the late protective signal is not driven by the early-period reversal pattern.*

# eTable 3. Subgroup analyses by age

Primary outcome (Composite Metastasis Endpoint) by age subgroups at 5-year follow-up.

| Subgroup | N per group | HR (95% CI) | P-value | P-interaction |
| --- | --- | --- | --- | --- |
| Age 18–49 years | 95 | 1·63 (0·74–3·55) | 0·218 | – |
| Age 50–64 years | 758 | 0·65 (0·39–1·10) | 0·106 | – |
| Age 65–74 years | 1,240 | 0·60 (0·41–0·87) | 0·007 | – |
| Age ≥75 years | 1,476 | 0·73 (0·49–1·10) | 0·136 | – |
| Total | **3,569** | – | – | – |
| Overall P-interaction | – | – | – | **0·092** |

*P-interaction tests for effect modification across age subgroups.*

# eTable 4. Subgroup analyses by race/ethnicity

Primary outcome (Composite Metastasis Endpoint) by race/ethnicity subgroups at 5-year follow-up. Patients with Unknown race (n=320 in the matched cohort, corresponding to the 8·9% Unknown Race category in Table 1) were not analysed as a separate subgroup because race is a sociocultural construct and 'Unknown' is not a meaningful demographic stratum for between-group comparison.

| Subgroup | N per group | HR (95% CI) | P-value | P-interaction |
| --- | --- | --- | --- | --- |
| white | 2,284 | 0·66 (0·49–0·89) | 0·006 | – |
| black or african american | 727 | 0·52 (0·32–0·84) | 0·006 | – |
| asian | 238 | 1·31 (0·67–2·54) | 0·425 | – |
| Total | **3,569** | – | – | – |
| Overall P-interaction | – | – | – | 0·066 |

# eTable 5. Subgroup analyses by breast cancer subtype

Primary outcome by breast cancer molecular subtype at 5-year follow-up. **Subtype classification was performed using the hierarchical algorithm described in eMethods 3** (Z17 receptor status codes plus subtype-defining therapy patterns).

| Subtype | N per group | HR (95% CI) | P-value | P-interaction |
| --- | --- | --- | --- | --- |
| HR-positive / HER2-negative | 2,257 | 0·78 (0·59–1·04) | 0·093 | – |
| HER2-positive (any HR) | 228 | 0·85 (0·44–1·65) | 0·629 | – |
| Triple-negative | 1,084 | 0·62 (0·40–0·98) | 0·037 | – |
| Total | **3,569** | – | – | – |
| Overall P-interaction | – | – | – | **0·594** |

*HR-positive = hormone receptor positive. HER2 = human epidermal growth factor receptor 2. Triple-negative = ER−/PR−/HER2−. The non-significant interaction test (p = 0·594) indicates no statistical evidence for differential effect by subtype; the TNBC point estimate should be interpreted in this light and in conjunction with the algorithmic subtyping limitations described in eMethods 3 §“Acknowledged Limitations”.*

# eTable 6. Subgroup analyses by SGLT2 inhibitor agent

Primary outcome by specific SGLT2 inhibitor agent at 5-year follow-up.

| Agent | N per group | HR (95% CI) | P-value | P-interaction |
| --- | --- | --- | --- | --- |
| Empagliflozin | 2,392 | 0·67 (0·51–0·90) | 0·006 | – |
| Dapagliflozin | 937 | 0·87 (0·54–1·43) | 0·588 | – |
| Canagliflozin | 240 | 0·90 (0·39–2·03) | 0·791 | – |
| Ertugliflozin | NA | NA | NA | – |
| Total | **3,569** | – | – | – |
| Overall P-interaction | – | – | – | **0·551** |

*Analyses performed separately for each SGLT2i agent versus non-SGLT2i users. Ertugliflozin excluded due to insufficient sample size.*

# eTable 7. E-value analysis for unmeasured confounding

E-values for primary and secondary outcomes (5-year follow-up).

| Outcome | SGLT2i N (events) | Non-SGLT2i N (events) | HR (95% CI) | E-value (point) | E-value (CI limit) |
| --- | --- | --- | --- | --- | --- |
| Composite Metastasis Endpoint* | 3,569 (297) | 3,569 (339) | 0·676 (0·532–0·859) | 2·32 | 1·60 |
| Lung Metastasis | 3,569 (39) | 3,569 (61) | 0·550 (0·351–0·863) | 3·04 | 1·59 |
| Liver Metastasis | 3,569 (29) | 3,569 (54) | 0·461 (0·278–0·765) | 3·76 | 1·94 |
| Brain Metastasis | 3,569 (26) | 3,569 (27) | 0·837 (0·460–1·523) | 1·68 | 1·00 |
| Bone Metastasis | 3,569 (55) | 3,569 (83) | 0·576 (0·394–0·843) | 2·87 | 1·66 |
| Bone Metastasis + SREs | 3,569 (91) | 3,569 (107) | 0·747 (0·546–1·020) | 2·01 | 1·00 |

*E-values ≥2·0 suggest moderate robustness; E-values ≥3·0 suggest strong robustness to unmeasured confounding. E-value for 95% CI represents the E-value for the confidence interval limit closest to the null (HR=1); a value of 1·00 indicates the confidence interval reaches the null (per VanderWeele & Ding, 2017).*

# eTable 8. Negative control outcomes (traditional)

Conditions without biological plausibility for differential association with SGLT2 inhibitor use. 5-year follow-up; matched cohorts (N = 3,569 per group).

| Outcome | ICD-10-CM | SGLT2i Events | Non-SGLT2i Events | HR (95% CI) | P-value |
| --- | --- | --- | --- | --- | --- |
| Traumatic fracture (non-pathological) | S02–S92 | 482 | 447 | 0·954 (0·838–1·085) | 0·475 |
| Kidney stone | N20–N23 | 243 | 245 | 0·894 (0·748–1·068) | 0·218 |

*Both NCOs show non-significant associations with confidence intervals crossing the null, supporting study validity and absence of systematic bias related to differential healthcare utilisation.*

# eTable 8b. Imaging-specific negative control outcomes

Conditions incidentally detected through the same abdominal and thoracic imaging modalities used for distant metastasis assessment, but without biological relationship to SGLT2 inhibitor pharmacology. Post-hoc analyses to interrogate imaging-driven detection bias. 5-year follow-up; matched cohorts (N = 3,569 per group).

| Outcome | ICD-10-CM | Imaging modality bias tested | SGLT2i Events | Non-SGLT2i Events | HR (95% CI) | P-value |
| --- | --- | --- | --- | --- | --- | --- |
| Thyroid nodule | E04·1 | Chest CT incidental finding | 281 | 251 | 1·024 (0·836–1·255) | 0·250 |
| Hepatic cyst | K76·89 | Abdominal CT/MRI incidental | 193 | 185 | 0·948 (0·745–1·205) | 0·977 |
| Renal cyst | N28·1 | Abdominal CT/MRI incidental | 233 | 203 | 1·026 (0·820–1·284) | 0·823 |

*All three imaging-specific NCOs returned null associations, arguing against differential imaging utilisation between cohorts as an explanation for the primary metastasis finding. If imaging-driven detection bias were operating, we would expect correspondingly different rates of these incidentally detected conditions; the observed null results refute this mechanism.*

# eTable 8c. Negative control exposure analyses (DPP-4 inhibitor and statin)

Two parallel 1:1 propensity-score-matched analyses substituting the index exposure to test (NCE-1) within-class antidiabetic confounding and (NCE-2) general chronic-medication confounding. Outcome: composite distant metastasis at 5 years. Eligibility, covariate set (with the respective index exposure removed), and analytic framework identical to the primary analysis.

| Exposure comparison | N per group | Composite metastasis HR (95% CI) | P-value | Inference |
| --- | --- | --- | --- | --- |
| **SGLT2i** (primary, for reference) | 3,569 | **0·676 (0·532–0·859)** | **0·001** | Significant protective association |
| **NCE-1: DPP-4 inhibitor** vs non-DPP-4 inhibitor (both excluding SGLT2i) | 1,328 | 1·128 (0·863–1·473) | 0·378 | Null – rejects within-class antidiabetic confounding |
| **NCE-2: Statin** vs non-statin (both excluding SGLT2i) | 4,273 | 0·903 (0·779–1·046) | 0·174 | Null – rejects general chronic-medication confounding |

*The null DPP-4 inhibitor and statin negative control exposure results, combined with the substantially more protective SGLT2i estimate, support drug-class specificity of the observed SGLT2i–metastasis association rather than a class-level antidiabetic effect or generic chronic-medication “healthy adherer” phenomenon.*

# eTable 9. SGLT2 inhibitor drug codes

| Drug Name | Brand Name(s) | RxNorm Code | ATC Code |
| --- | --- | --- | --- |
| Dapagliflozin | Farxiga | 1488564 | A10BK01 |
| Empagliflozin | Jardiance | 1545653 | A10BK03 |
| Canagliflozin | Invokana | 1373458 | A10BK02 |
| Ertugliflozin | Steglatro | 1992672 | A10BK04 |
| SGLT2i Class | – | – | A10BK |

# eTable 10. Outcome definitions and ICD-10 codes

| Outcome | Definition | ICD-10 / CPT / Medication Codes |
| --- | --- | --- |
| **Primary Outcomes** |  |  |
| Composite Metastasis Endpoint* | Distant metastasis: secondary malignant neoplasm of respiratory and digestive organs (C78), or other and unspecified sites (C79) | ICD-10-CM: C78, C79 |
| Lung Metastasis | Secondary malignant neoplasm of lung, mediastinum, pleura, or other respiratory organs | ICD-10-CM: C78·0, C78·1, C78·2, C78·3 |
| Liver Metastasis | Secondary malignant neoplasm of liver and intrahepatic bile duct | ICD-10-CM: C78·7 |
| Brain Metastasis | Secondary malignant neoplasm of brain and cerebral meninges | ICD-10-CM: C79·3 |
| Bone Metastasis | Secondary malignant neoplasm of bone and bone marrow | ICD-10-CM: C79·5 |
| **Secondary Outcomes** |  |  |
| Skeletal-Related Events | Bone metastasis, pathological fracture in neoplastic disease, pathological fracture NEC, cord compression, or nerve root/plexus compression | ICD-10-CM: C79·5, M84·5, M84·4, G95·2, G55 |
| All-Cause Mortality | Death from any cause during follow-up | TriNetX death record |

*Composite Metastasis Endpoint = Distant Metastasis, combining C78 (secondary malignant neoplasm of respiratory and digestive organs) and C79 (secondary malignant neoplasm of other and unspecified sites). Lung, liver, brain, and bone metastasis are individual components of the composite endpoint. ICD-10-CM = International Classification of Diseases, 10th Revision, Clinical Modification. RxNorm = RxNorm concept unique identifier; HCPCS = Healthcare Common Procedure Coding System; ATC = Anatomical Therapeutic Chemical Classification. NEC = not elsewhere classified. Metastasis ascertainment in this study relied on the diagnosis codes above; we did not require concurrent procedure-code documentation (e.g., CPT 71250–71270 chest CT, 74176–74178 abdomen/pelvis CT/MRI, 70551–70553 brain MRI) as additional positive ascertainment because TriNetX clinical-fact density for imaging procedures is variable across contributing healthcare organisations and could introduce between-cohort imbalance in apparent outcome rates if used as a gating criterion.*

# eTable 11. Propensity score matching covariates

Complete list of 26 covariates included in the propensity score model, assessed during the baseline period (before index event).

| Category | Variables |
| --- | --- |
| Demographics (4) | Age at index, Sex (Female), Race (white, black or african american, asian, Unknown), Ethnicity (hispanic/non-hispanic) |
| Cardiovascular (6) | Essential hypertension (I10), Heart failure (I50), Ischaemic heart disease (I20–I25), Chronic kidney disease (N18), Cerebrovascular diseases (I60–I69), Dyslipidaemia (E78) |
| Metabolic (2) | Overweight and obesity (E66), HbA1c level |
| Cancer Treatment (3) | Antineoplastic chemotherapy (Z51·11), Antineoplastic hormones, Trastuzumab |
| Antidiabetic Medications (7) | Biguanides (Metformin), Sulfonylureas, Alpha-glucosidase inhibitors, Thiazolidinediones, DPP-4 inhibitors, GLP-1 receptor agonists, Insulin |
| Cardiovascular Medications (3) | Beta-blockers, ACE inhibitors, ARBs |
| Laboratory Values (1) | HbA1c (categorical: <7% vs ≥7%) |

*All covariates assessed at baseline (before index event). SMD <0·1 for all variables after matching indicates adequate balance.*

# eTable 12. Gene set definitions for pathway activity analysis

Complete gene set definitions used in the single-sample gene set enrichment analysis (ssGSEA). Hallmark gene sets were obtained from MSigDB v2023·2 (Broad Institute) via the *msigdbr* R package. Custom gene sets were assembled from published literature as indicated. The WARBURG_INDEX is a composite index computed from the glycolysis and OXPHOS ssGSEA scores and does not have a separate gene list.

| Gene Set | Source | n Genes | n Matched† | Key Component Genes (representative) |
| --- | --- | --- | --- | --- |
| Glycolysis | HALLMARK_GLYCOLYSIS | 200 | 181 | HK1, HK2, PFKL, PFKM, PKM, LDHA, LDHB, ENO1, GAPDH, PGK1, ALDOA, TPI1, GPI, SLC2A1, SLC2A3 |
| Oxidative Phosphorylation | HALLMARK_OXIDATIVE_PHOSPHORYLATION | 200 | 155 | NDUFA1–NDUFV3, SDHA/B/C/D, UQCRC1/2, COX4I1, COX5A, ATP5F1A/B/C, CYC1, CYCS |
| Fatty Acid Metabolism | HALLMARK_FATTY_ACID_METABOLISM | 158 | 132 | ACOX1, CPT1A, CPT2, FASN, SCD, ACACA, ACLY, HADHA, HADHB, ACADM, ACADL |
| Hypoxia | HALLMARK_HYPOXIA | 200 | 180 | HIF1A, VEGFA, SLC2A1, LDHA, PGK1, ENO1, ALDOA, CA9, BNIP3, PDK1, EGLN1 |
| mTORC1 Signaling | HALLMARK_MTORC1_SIGNALING | 200 | 171 | RPS6KB1, EIF4EBP1, MTOR, RPTOR, SLC7A5, SLC3A2, SCD, ACLY, SQSTM1, ATF4 |
| PI3K/AKT/mTOR | HALLMARK_PI3K_AKT_MTOR_SIGNALING | 105 | 84 | PIK3CA, PIK3CB, AKT1, AKT2, MTOR, PTEN, TSC1, TSC2, RHEB, PDPK1 |
| EMT | HALLMARK_EPITHELIAL_MESENCHYMAL_TRANSITION | 200 | 194 | VIM, CDH2, SNAI1, SNAI2, TWIST1, ZEB1, ZEB2, FN1, MMP2, ITGA5, COL1A1 |
| Angiogenesis | HALLMARK_ANGIOGENESIS | 36 | 33 | VEGFA, KDR, FLT1, PECAM1, ANGPT1, ANGPT2, TEK, NRP1, FGF2, PDGFRB |
| Inflammatory Response | HALLMARK_INFLAMMATORY_RESPONSE | 200 | 181 | IL6, IL1B, CXCL8, TNF, CCL2, CCL5, ICAM1, VCAM1, SELE, PTGS2, NFKBIA |
| TNFα/NFκB Signaling | HALLMARK_TNFA_SIGNALING_VIA_NFKB | 200 | 190 | NFKB1, RELA, TNFAIP3, BIRC3, CCL2, CXCL1, IL6, ICAM1, TRAF1, NFKBIA |
| Glutamine Metabolism | Custom (literature-derived) | 34 | 29 | GLS, GLS2, SLC1A5, SLC38A1, SLC38A2, SLC7A5, SLC7A11, GOT1, GOT2, GLUD1, MYC, CAD, DHODH |
| AMPK Signaling | Custom (literature-derived) | 43 | 34 | PRKAA1, PRKAA2, PRKAB1, PRKAG1, STK11, CAMKK2, STRADA, CAB39, TSC2, ULK1, PFKFB3, PPARGC1A, SIRT1 |
| Insulin/IGF Signaling | Custom (literature-derived) | 51 | 44 | INSR, IGF1R, IRS1, IRS2, IGF1, IGF2, IGFBP1–IGFBP7, GRB2, SOS1, PIK3CA, AKT1, MTOR, PTEN, SLC2A1 |
| RANKL/Osteoclast | Custom (literature-derived) | 43 | 41 | TNFSF11 (RANKL), TNFRSF11A (RANK), TNFRSF11B (OPG), NFATC1, CTSK, ACP5, MMP9, TRAF6, CSF1, RUNX2, SPP1 |
| Warburg Index | Composite: Glycolysis – OXPHOS ssGSEA scores | N/A | N/A | Computed per sample as difference between glycolysis and OXPHOS enrichment scores |

† n Matched indicates the number of genes from each gene set that were present in the GSE110590 expression matrix after gene identifier mapping and low-variance filtering. Hallmark gene set sizes reflect MSigDB v2023·2 definitions accessed via the *msigdbr* R package (species: *Homo sapiens*, category: H).

*Abbreviations: MSigDB, Molecular Signatures Database; ssGSEA, single-sample gene set enrichment analysis; OXPHOS, oxidative phosphorylation; AMPK, AMP-activated protein kinase; IGF, insulin-like growth factor; RANKL, receptor activator of nuclear factor kappa-B ligand; EMT, epithelial–mesenchymal transition; TNFα, tumour necrosis factor alpha; NFκB, nuclear factor kappa-light-chain-enhancer of activated B cells.*

# eTable 13. Complete ssGSEA results: paired pathway activity changes between primary tumour and distant metastasis (GSE110590)

Paired within-patient changes in pathway activity scores (ssGSEA) between primary breast tumour and matched distant metastasis, stratified by metastatic site. Effect sizes are reported as paired Cohen’s *d* with approximate 95% confidence intervals (*d* ± 1·96/√n). P values were derived from Wilcoxon signed-rank tests. FDR-adjusted P values were calculated using the Benjamini-Hochberg procedure within each organ site (15 comparisons per site). Positive Cohen’s *d* indicates higher pathway activity in the metastasis relative to the primary tumour.

| Pathway | Liver (n = 10) Cohen’s d (95% CI) / FDR | Lung (n = 10) Cohen’s d (95% CI) / FDR | Brain (n = 6) Cohen’s d (95% CI) / FDR | Bone (n = 4) Cohen’s d (95% CI) / FDR |
| --- | --- | --- | --- | --- |
| **Core Metabolic Pathways** |  |  |  |  |
| Glycolysis | +1·49 (+0·87, +2·11) ★ / 0·029 | +1·06 (+0·44, +1·68) † / 0·183 | +2·50 (+1·70, +3·30) / 0·134 | +0·33 (−0·65, +1·31) / 0·312 |
| Oxidative Phosphorylation | +1·34 (+0·72, +1·96) ★ / 0·034 | +0·87 (+0·25, +1·49) / 0·226 | +1·87 (+1·07, +2·67) / 0·134 | +0·47 (−0·51, +1·45) / 0·312 |
| Fatty Acid Metabolism | +1·25 (+0·63, +1·87) ★ / 0·034 | +0·80 (+0·18, +1·42) / 0·210 | +1·56 (+0·76, +2·36) / 0·180 | +0·17 (−0·81, +1·15) / 0·781 |
| Hypoxia | +1·34 (+0·72, +1·96) ★ / 0·034 | +1·17 (+0·55, +1·79) ★ / 0·029 | +1·55 (+0·75, +2·35) † / 0·134 | +0·08 (−0·90, +1·06) / 0·781 |
| mTORC1 Signaling | +0·93 (+0·31, +1·55) ★ / 0·037 | +0·86 (+0·24, +1·48) † / 0·098 | +1·45 (+0·65, +2·25) / 0·134 | +0·75 (−0·23, +1·73) / 0·417 |
| **Signaling Pathways** |  |  |  |  |
| PI3K/AKT/mTOR | +0·60 (−0·02, +1·22) / 0·074 | +1·14 (+0·52, +1·76) ★ / 0·029 | +1·10 (+0·30, +1·90) / 0·180 | −0·04 (−1·02, +0·94) / 0·875 |
| Glutamine Metabolism | +0·86 (+0·24, +1·48) / 0·074 | +0·42 (−0·20, +1·04) / 0·738 | +0·65 (−0·15, +1·45) / 0·156 | +0·37 (−0·61, +1·35) / 0·562 |
| AMPK Signaling | −0·93 (−1·55, −0·31) † / 0·062 | −0·75 (−1·37, −0·13) / 0·300 | −0·83 (−1·63, −0·03) / 0·180 | −0·25 (−1·23, +0·73) / 0·312 |
| Insulin/IGF Signaling | −0·47 (−1·09, +0·15) / 0·074 | +0·03 (−0·59, +0·65) / 0·922 | −0·76 (−1·56, +0·04) / 0·156 | −0·47 (−1·45, +0·51) / 0·312 |
| **Microenvironment Pathways** |  |  |  |  |
| RANKL/Osteoclast | −0·95 (−1·57, −0·33) ★ / 0·029 | −0·01 (−0·63, +0·61) / 0·906 | −1·07 (−1·87, −0·26) † / 0·134 | −0·15 (−1·13, +0·83) / 0·875 |
| EMT | −0·63 (−1·25, −0·01) † / 0·073 | −0·06 (−0·68, +0·56) / 0·906 | −0·65 (−1·45, +0·15) / 0·180 | −0·02 (−1·00, +0·96) / 0·875 |
| Angiogenesis | +0·21 (−0·41, +0·83) / 0·492 | +0·60 (−0·02, +1·22) / 0·193 | −0·39 (−1·19, +0·41) / 0·562 | +0·50 (−0·48, +1·48) / 0·312 |
| Inflammatory Response | −0·69 (−1·31, −0·07) ★ / 0·037 | +0·16 (−0·46, +0·78) / 0·906 | −1·24 (−2·04, −0·44) / 0·134 | −0·66 (−1·64, +0·32) / 0·312 |
| TNFα/NFκB Signaling | −0·42 (−1·04, +0·20) / 0·140 | +0·19 (−0·43, +0·81) / 0·906 | −0·89 (−1·69, −0·09) † / 0·134 | −0·55 (−1·53, +0·43) / 0·417 |
| **Composite Index** |  |  |  |  |
| Warburg Index | −1·17 (−1·79, −0·55) ★ / 0·034 | −0·63 (−1·25, −0·01) / 0·322 | −1·08 (−1·88, −0·28) / 0·469 | −0·39 (−1·37, +0·59) / 0·417 |

*★ FDR <0·05; † nominal P <0·05 (FDR ≥0·05). Abbreviations: ssGSEA, single-sample gene set enrichment analysis; FDR, false discovery rate; CI, confidence interval; EMT, epithelial–mesenchymal transition; OXPHOS, oxidative phosphorylation.*

# eTable 14. All-cause mortality in the matched cohort

All-cause mortality at 1-, 3-, and 5-year follow-up windows in the same 1:1 propensity-score-matched cohort used for the primary metastasis analysis. Matched cohort N = 3,569 per group at index; per-window at-risk N decreases over follow-up due to censoring and administrative truncation. Mortality rates are computed against per-window at-risk denominators.

| Follow-up | SGLT2i mortality % | Non-SGLT2i mortality % | Risk Ratio (95% CI) | Hazard Ratio (95% CI) | Log-Rank P |
| --- | --- | --- | --- | --- | --- |
| 1-year | 1·06% | 1·06% | 1·000 (0·614–1·628) | 1·143 (0·700–1·866) | 0·593 |
| 3-year | 5·59% | 5·86% | 0·954 (0·765–1·190) | 1·199 (0·954–1·506) | 0·119 |
| 5-year | 8·81% | 9·24% | 0·954 (0·808–1·126) | 0·986 (0·814–1·194) | 0·885 |

*All-cause mortality was not significantly different between SGLT2 inhibitor users and non-users at any follow-up window. The absence of differential mortality argues against competing risk as a meaningful source of bias in the cause-specific distant-metastasis estimate; under null mortality differential, cause-specific and subdistribution hazards converge.*

# eTable 15. Geographic distribution of TriNetX healthcare organisations contributing to the eligible cohort

Geographic distribution of the 18 317 eligible patients identified prior to propensity score matching, reported by the location of the contributing healthcare organisation (HCO). Of the 171 healthcare organisations in the TriNetX Global Collaborative Network, 95 contributed at least one eligible patient to this cohort. US-based patients are stratified by US Census Bureau Division; international patients are stratified by TriNetX regional sub-network. Counts were derived from the TriNetX platform Cohort Analytics export (run date 2026-04-16). Because the TriNetX network is federated, individual HCO names and country-level attribution within international sub-regions are not released by the platform.

| Region | Patients, n | Percent | States / Areas Included (examples) |
| --- | --- | --- | --- |
| **United States – by US Census Division** |  |  |  |
| South Atlantic | 4,082 | 22·3% | FL, GA, NC, SC, VA, WV, DC, DE, MD |
| Middle Atlantic | 3,917 | 21·4% | NJ, NY, PA |
| East North Central | 1,862 | 10·2% | IL, IN, MI, OH, WI |
| West North Central | 1,730 | 9·4% | IA, KS, MN, MO, NE, ND, SD |
| West South Central | 1,125 | 6·1% | AR, LA, OK, TX |
| East South Central | 738 | 4·0% | AL, KY, MS, TN |
| **US Subtotal** | **13,454** | **73·4%** |  |
| **International – by TriNetX Region** |  |  |  |
| Asia–Pacific (APAC) | 1,950 | 10·6% | Multiple countries via TriNetX Global Network |
| Europe, Middle East & Africa (EMEA) | 1,373 | 7·5% | Multiple countries via TriNetX Global Network |
| Latin America | 720 | 3·9% | Multiple countries via TriNetX Global Network |
| Other international HCOs | 820 | 4·5% | Multiple countries via TriNetX Global Network |
| **International Subtotal** | **4,863** | **26·6%** |  |
| **Total Eligible Cohort** | **18,317** | **100·0%** |  |

Abbreviations: HCO, healthcare organisation; APAC, Asia–Pacific; EMEA, Europe / Middle East / Africa. Percentages are rounded to one decimal place and may not sum exactly to 100%.

# eFigure 1. Hypothesised dual mechanism of SGLT2 inhibitor anti-metastatic protection in breast cancer (conceptual framework)

This conceptual schematic illustrates two hypothesised pathways by which SGLT2 inhibitors may reduce distant metastasis risk, framed at the level of inference from this study's observational findings, prior literature, and the exploratory paired transcriptomic analysis (GSE110590). No direct experimental evidence supporting either mechanism is presented in this study. Dashed arrows denote hypothesised – rather than established – causal linkages.

Zone 1: Hypothesised mechanistic pathways. Mechanism A (left; Hypothesis A – Local Metabolic Mismatch) proposes that SGLT2 inhibitor–induced reduction in systemic glucose availability creates an energy supply–demand mismatch at metabolically hyperactive metastatic niches, impairing metastatic colonisation. Mechanism B (right; Hypothesis B – Systemic Effects) proposes that SGLT2 inhibitor activation of AMPK and reduction of circulating insulin / IGF-1 (both literature-supported), together with elevated ketone bodies and RANKL-mediated osteoclast suppression, contribute to systemic restraint of metastatic establishment, particularly in bone.

Zone 2: Organ-specificity matrix (descriptive – not experimental ratings). A two-dimensional descriptive mapping of organ-specific metastasis on axes of relative metabolic demand (inferred from the paired transcriptomic gradient; eTable 13) and modelled drug exposure. Liver and lung metastases (red, blue) align with high metabolic demand and significant clinical association – consistent with Mechanism A. Bone (green) shows significant clinical association despite low local metabolic demand – consistent with Mechanism B (decoupled from local metabolism). Brain (orange) was non-significant and underpowered, with limited blood–brain-barrier penetration of SGLT2 inhibitors as an additional caveat. Positions are descriptive only and were not derived from quantitative effect-size scoring.

Zone 3: Clinical correspondence (descriptive only). Summary of organ-specific clinical association strength: liver (strongest); lung (significant); brain (non-significant, sample-size-limited); bone (significant, via systemic pathway).

Caveat. These pathways are hypothesis-generating and have not been experimentally validated within this study. Mechanism arrows depict proposed, not established, causal links. See manuscript Methods and Discussion for the observational design and analytic limitations.

*Abbreviations: SGLT2, sodium–glucose cotransporter 2; AMPK, AMP-activated protein kinase; IGF-1, insulin-like growth factor 1; RANKL, receptor activator of nuclear factor kappa-B ligand; BBB, blood–brain barrier.*

# eFigure 2. Aalen-Johansen cumulative incidence functions for distant metastasis and all-cause mortality

**Legend.** Aalen-Johansen non-parametric cumulative incidence functions for distant metastasis (Panel A) and all-cause mortality (Panel B), treating each event as a competing risk for the other. Curves reflect the unmatched eligible cohorts because the federated TriNetX platform does not support cumulative-incidence-function estimation after propensity-score matching; shaded regions are 95% confidence intervals. Five-year cumulative incidence values: distant metastasis 5·21% (SGLT2i) versus 7·63% (non-SGLT2i); all-cause mortality 2·76% (SGLT2i) versus 5·69% (non-SGLT2i). The simultaneous reduction in both endpoints under SGLT2 inhibitor exposure is incompatible with a competing-risk explanation for the observed protective metastasis association: a competing-risk artifact (i.e., differential survival inflating apparent metastasis incidence in the comparator arm) would produce opposing directions for the two CIFs, not the same direction.

*Abbreviation: CIF, cumulative incidence function.*

*End of Supplementary Materials.*
